# Nitric Oxide Production by Necrotrophic Pathogen *Macrophomina phaseolina* and the Host Plant in Charcoal Rot Disease of Jute: Complexity of the Interplay between Necrotroph–Host Plant Interactions

**DOI:** 10.1371/journal.pone.0107348

**Published:** 2014-09-10

**Authors:** Tuhin Subhra Sarkar, Pranjal Biswas, Subrata Kumar Ghosh, Sanjay Ghosh

**Affiliations:** 1 Department of Biochemistry, University of Calcutta, Kolkata, India; 2 Centre for Research in Nanoscience and Nanotechnology (CRNN), University of Calcutta, Kolkata, India; 3 Former head, Division of Crop Protection, Central Research Institute for Jute and Allied Fibres (CRIJAF), Kolkata, India; University of Edinburgh, United Kingdom

## Abstract

*M. phaseolina*, a global devastating necrotrophic fungal pathogen causes charcoal rot disease in more than 500 host plants. With the aim of understanding the plant-necrotrophic pathogen interaction associated with charcoal rot disease of jute, biochemical approach was attempted to study cellular nitric oxide production under diseased condition. This is the first report on *M. phaseolina* infection in *Corchorus capsularis* (jute) plants which resulted in elevated nitric oxide, reactive nitrogen species and S nitrosothiols production in infected tissues. Time dependent nitric oxide production was also assessed with 4-Amino-5-Methylamino-2′,7′-Difluorofluorescein Diacetate using single leaf experiment both in presence of *M. phaseolina* and xylanases obtained from fungal secretome. Cellular redox status and redox active enzymes were also assessed during plant fungal interaction. Interestingly, *M. phaseolina* was found to produce nitric oxide which was detected *in vitro* inside the mycelium and in the surrounding medium. Addition of mammalian nitric oxide synthase inhibitor could block the nitric oxide production in *M. phaseolina*. Bioinformatics analysis revealed nitric oxide synthase like sequence with conserved amino acid sequences in *M. phaseolina* genome sequence. In conclusion, the production of nitric oxide and reactive nitrogen species may have important physiological significance in necrotrophic host pathogen interaction.

## Introduction

Plants have developed a diversity of defense mechanisms to protect themselves against pathogen colonization. Basal defense system plays an important role in plant immunity. With the help of a much less-specific recognition system plants identify pathogen associated molecular patterns to prevent invasion and to restrict pathogen growth [1]. In response to pathogens that overcome basal defense, plants have evolved resistance proteins that promote inducible defense responses as characterized by hypersensitive response (HR) upon pathogen recognition. Cell death during HR compels invading biotrophic pathogen to limit pathogen growth because biotrophic pathogen utilizes nutrients from living host cells. They cannot survive in dead material. But in case of necrotrophic pathogen, host cell death may be beneficial for its growth and pathogenesis.

Cell death during HR is thought to be dependent on balanced production of nitric oxide (NO) and reactive oxygen species (ROS) [2]. Recent studies indicated that the levels of NO in plant cells, fungal mycelium and interaction medium might have important consequences in the success of the fungal infection. The production of NO in germinating conidia and developing mycelium was detected by van Baarlen [3] in *Botrytis cinerea*, a necrotrophic fungus, through the use of fluorescent probes. In parallel, the production of NO was detected in *B. cinerea in vitro* by mass spectrometry [4]. Strong NO generation was detected using 4, 5-diaminofluorescein diacetate (DAF-2 DA) during the *B. cinerea* colonization of pelargonium leaves [5]. NO also plays an important role in signalling in fungi. The application of external NO to the necrotrophic fungus *Colletotrichum coccodes* was found to delay spore germination, whereas treatment with NO scavengers stimulated spore germination [6]. In the biotrophic fungus *Blumeria graminis*, application of either an NO scavenger or a mammalian Nitric oxide synthase (NOS) inhibitor affected appressorium formation [7], [8]. However, there is still little information available regarding the role of NO in necrotrophic pathogen.

*M. phaseolina* is a global devastating necrotrophic fungal pathogen. It causes the charcoal rot disease. It infects more than 500 plant hosts [9], [10] including major food crops [11], pulse crops [12], [13], fiber crops (jute [14], cotton [15]) and oil crops [9]. Though it has a wide host range, *Macrophomina* is a monotypic genus. *M. phaseolina* is an anamorphic fungus in the phylum ascomycete, family Botryosphaeriaceae [16]. This pathogen can result in severe crop losses. For example, this pathogen accounted for a total yield loss of $173.80 million in the United States during 2002 [17]. In India and Bangladesh also, charcoal rot disease caused substantial loss of fiber yield of jute [10], [18].

*M. phaseolina* has been known as a necrotrophic fungus for a long time, still studies on the biosynthetic pathway for NO production by *M. phaseolina*, and its implications for plant infection, have not been investigated before. With the aim of understanding the plant-necrotrophic pathogen interaction associated with charcoal rot disease of jute, biochemical approach was attempted to study cellular NO production under diseased condition. Here we have for the first time demonstrated that *M. phaseolina* infection in *C. capsularis* (jute) plants results in elevated NO production in infected tissues. Furthermore, we have provided evidences of NO production in *M. phaseolina.* The presence of NOS like sequence in *M. phaseolina* genome has opened up new areas of research regarding its evolutionary significance among the microorganisms.

## Results

### Induction of charcoal rot disease during *C. capsularis* JRC 412- *M. phaseolina* (strain R9) interaction

**Figure 1:**
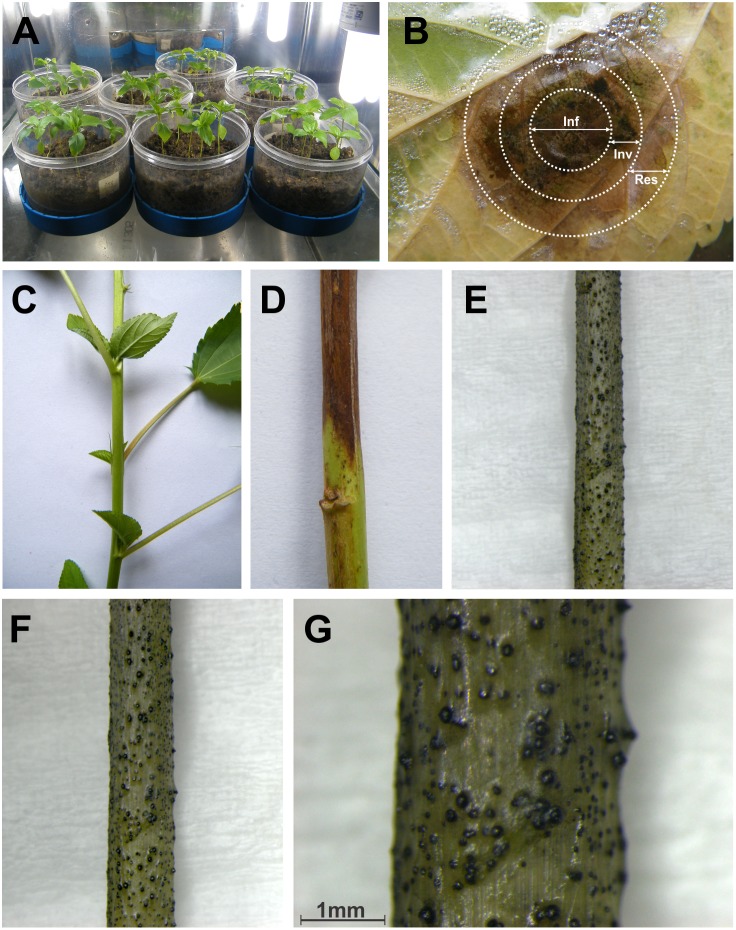
Different stages of *M. phaseolina* infected jute (*C. capsularis*) plant. Jute seedlings were grown under controlled conditions (A), Three approximate distinctive areas are shown in infected leaf (B): infected area (Inf), invaded area (Inv), responsive area (Res), (C) represents healthy jute plant, (D) represents dark brown lesion of infected stem, (E), (F) and (G) represent symptoms of charcoal-rot disease in infected stem of jute.

Previous studies on screening for stem rot tolerant jute accessions were carried out at three different locations namely Central Research Institute of Jute & Allied Fibres (CRIJAF), Barrackpore, Budbud in West Bengal and Sorbhog in Assam which revealed the field tolerance of nine accessions of *C. capsularis* [19]. Among those, a cultivated variety, JRC 412 showed susceptibility. In the present study, susceptible variety JRC 412 was used for all the experiments. JRC 412 was grown under a polythene shed, where the environment was maintained at an ambient level. The plants were raised in autoclaved transparent polycarbonate pots (containing coarse, acid washed, neutral pH sand with autoclaved soilrite) (Figure 1A). Adequate measures were taken to protect the plants from biotic and abiotic stresses. To induce charcoal rot disease, fungal mycelia were placed on the upper surface of jute leaves following leaf inoculation method. Disease lesions were prominent on stem at 20 days post inoculation (Figure 1B, D, E, F, and G). A greyish-black appearance was observed in the subepidarmal tissues of the stem (Figure 1D). Such discolouration was visible at nodes as profuse small, black, randomly distributed specks.

Based on the hyphal elongation of *M. phaseolina* stained with Lactophenol Cotton Blue as observed under microscope, disease lesions of plant cells were divided into three areas as shown in Figure 1B. These areas were classified as (i) Infected areas which encompassed the centre of lesions where massive hyphae and micro sclerotia were observed due to infection. (ii) Invaded areas included edge of the lesions where the apical part of hyphae was prominent along with the dead cells. (iii) Response areas were actually the regions adjacent to lesion where living cells were found. Dark browning of the adjacent tissues was observed after 20 days post inoculation in the susceptible variety (Figure 1D). Foliar symptoms gradually progressed from top of the plant to downwards. Leaves of infected plants remained smaller than normal and subsequently turned yellow prior to wilting. Similar results were observed in case of stem inoculation where browning of the adjacent tissues was observed indicating the progression of charcoal rot disease.

**Figure 2:**
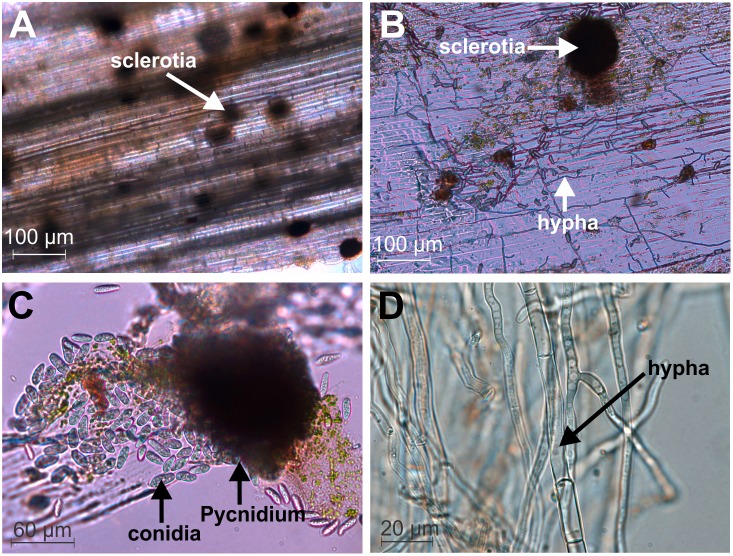
Morphological characteristics of *M. phaseolina*. Longitudinal sections of *M. phaseolina* infected Jute stem showing sclerotia (A), hyphal network (B). Light micrograph of globose pycnidia and conidia along with released conidia of *M. phaseolina* from the pycnidium (C). Aerial hyphae of *M. phaseolina* (D).

Figure 2 represents the distribution of micro-sclerotia in the vascular tissues and in the pith or central part of the infected stem. The infected mature and dry pods were found to be covered with black bodies (micro-sclerotia). Micro-sclerotia remained concentrated in some part of the infected tissues (Figure 2A, B). It was also distributed in a scattered manner in some infected areas. Pycnidium was found to contain numerous conidia as evidenced in microscopically observed infected tissue sections (Figure 2C). Figure 2D represents septed mycelia of *M. phaseolina*.

### Induction of NO, RNS and RSNO during *C. capsularis* JRC 412- *M. phaseolina* interaction

**Figure 3:**
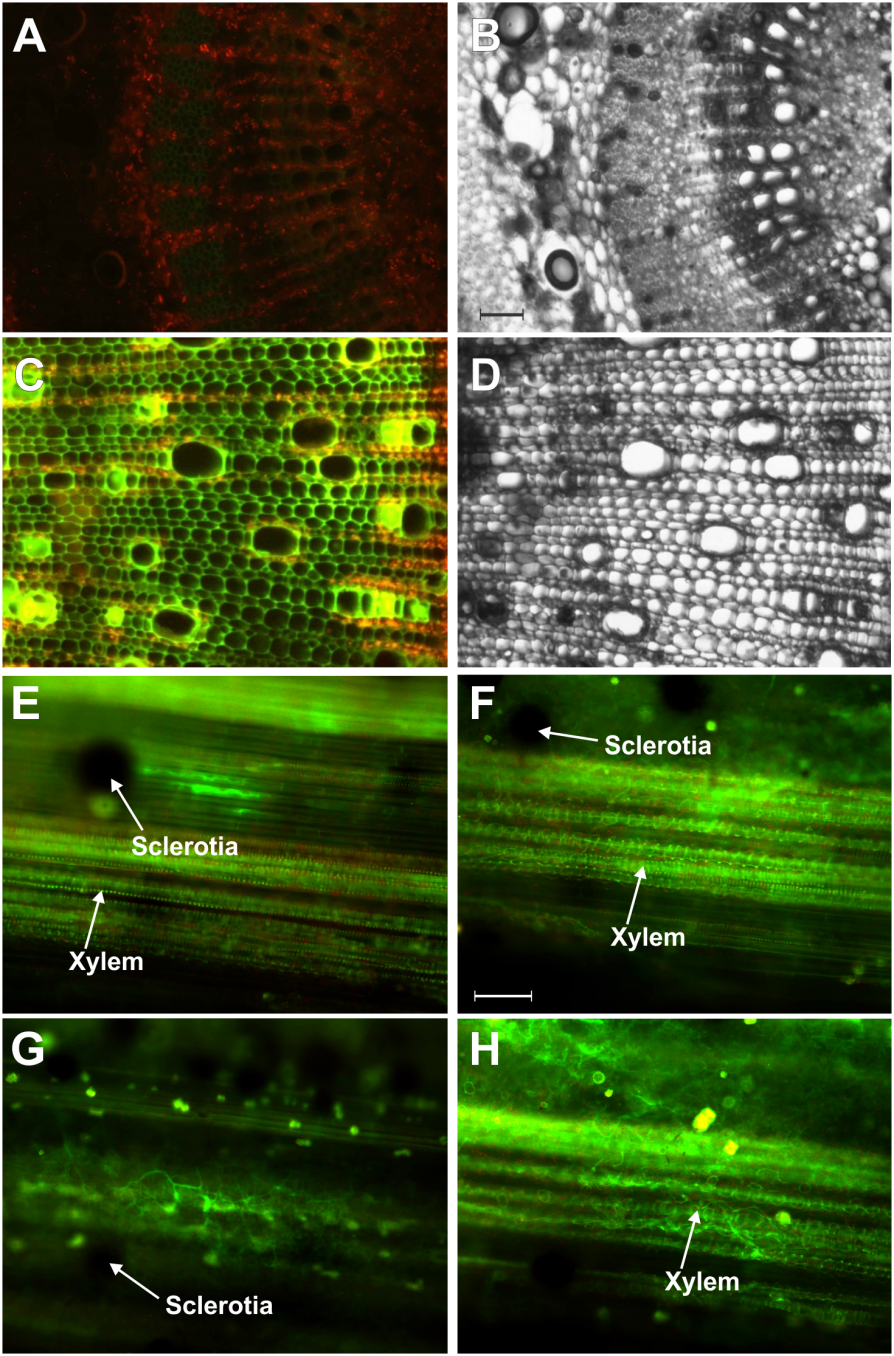
Detection of NO in *M. phaseolina* infected Jute stem stained with DAF FM-DA by fluorescence microscopy. Images represent cross sections (A–D) and longitudinal sections (E–H) of jute stem showing the bright green fluorescence corresponding to NO, bar = 60 µm (F). The red colour corresponds to the autofluorescence. (A) represents control stem cross section, (C) represents infected stem cross section, (B) and (D) are the corresponding bright fields respectively. Bar = 250 µm (B). Figures are representative of at least six independent experiments.

**Figure 4:**
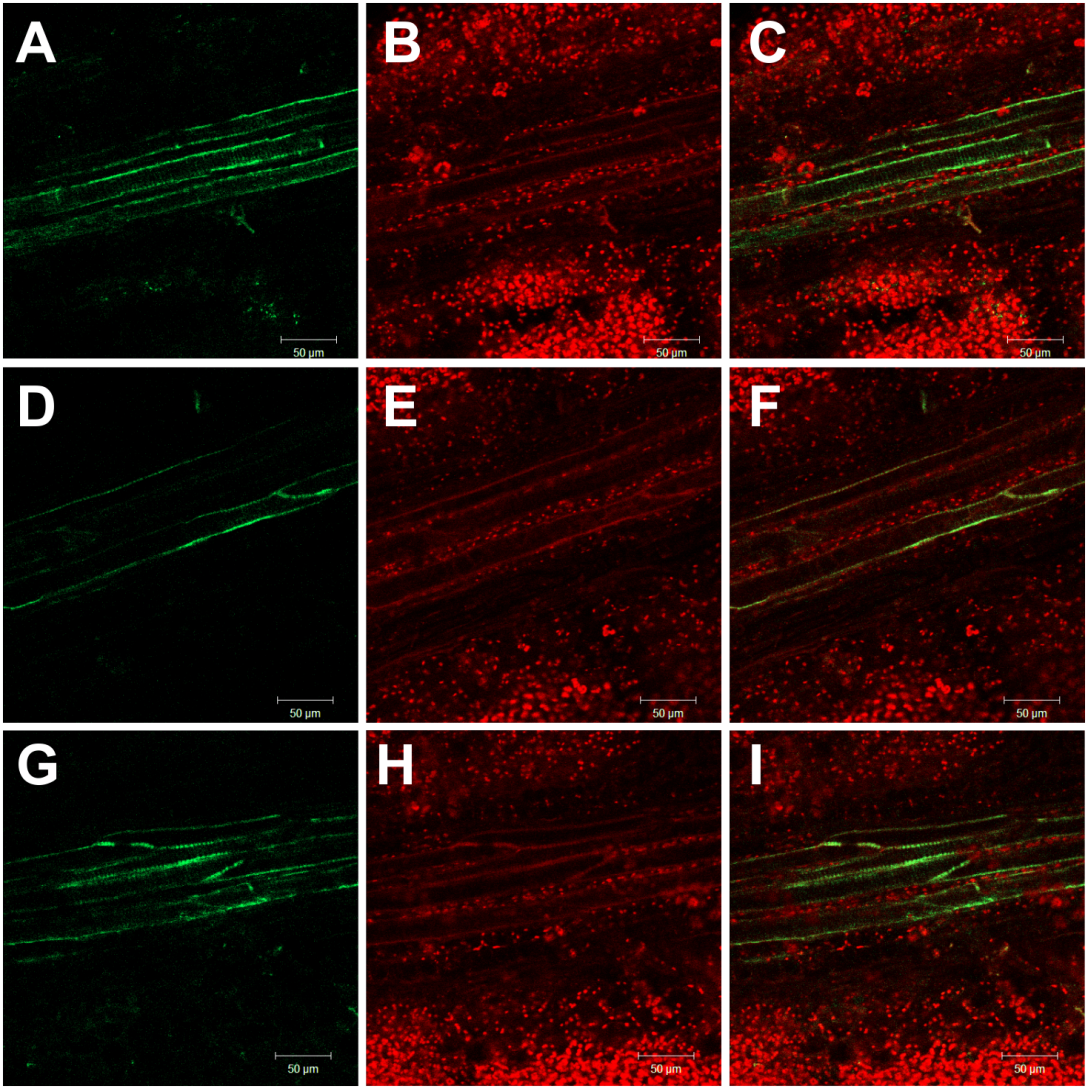
Detection of NO in longitudinal sections of mid rib portion of *M. phaseolina* infected Jute leaf. Leaf sections were stained with DAF FM-DA showing the presence of NO as bright green fluorescence (A, D and G). Red colour corresponded to chlorophyll auto fluorescence (B, E and H). Merge images represent both NO and autofluorescence (C, F and I).

To evaluate NO, ROS and RNS induction in susceptible variety of *C. capsularis* JRC 412 upon infection with *M. phaseolina*, both control and infected tissues with visible symptoms were taken for microscopic analysis. NO and RNS were detected by DAF-FM DA (4-Amino-5-Methylamino-2′,7′-Difluorofluorescein Diacetate) and DHR 123 (Dihydrorhodamine 123) mediated fluorescence respectively. Appearance of bright green fluorescence in the infected stem sections of susceptible variety of *C. capsularis* JRC 412 indicated NO specific fluorescence with DAF-FM DA (Figure 3 C, E, F, G, H). Figure 4 represents longitudinal cross section of infected leaf showing NO specific fluorescence with DAF-FM DA. NO specific fluorescence was found to be accumulated in the vascular bundle regions which indicated its systemic circulation in the infected area. Interestingly, NO specific fluorescence was found to be absent in control tissue sections (Figure 3A) which indicated that induction of NO occurred under diseased conditions only. Marked accumulation of NO was found in the response areas adjacent to the infection zone (Figure 3E, F, G, H). NO specific fluorescence was prominent in the vascular bundle region containing invaded mycelium and micro-sclerotia. The inducible fluorescence was scavenged by 2-(4-carboxyphenyl)-4,4,5,5-tetramethylimidazoline-1-oxyl-3-oxide (cPTIO), an NO scavenger (Figure S1), indicating that DAF- FM detects specifically NO.

**Figure 5:**
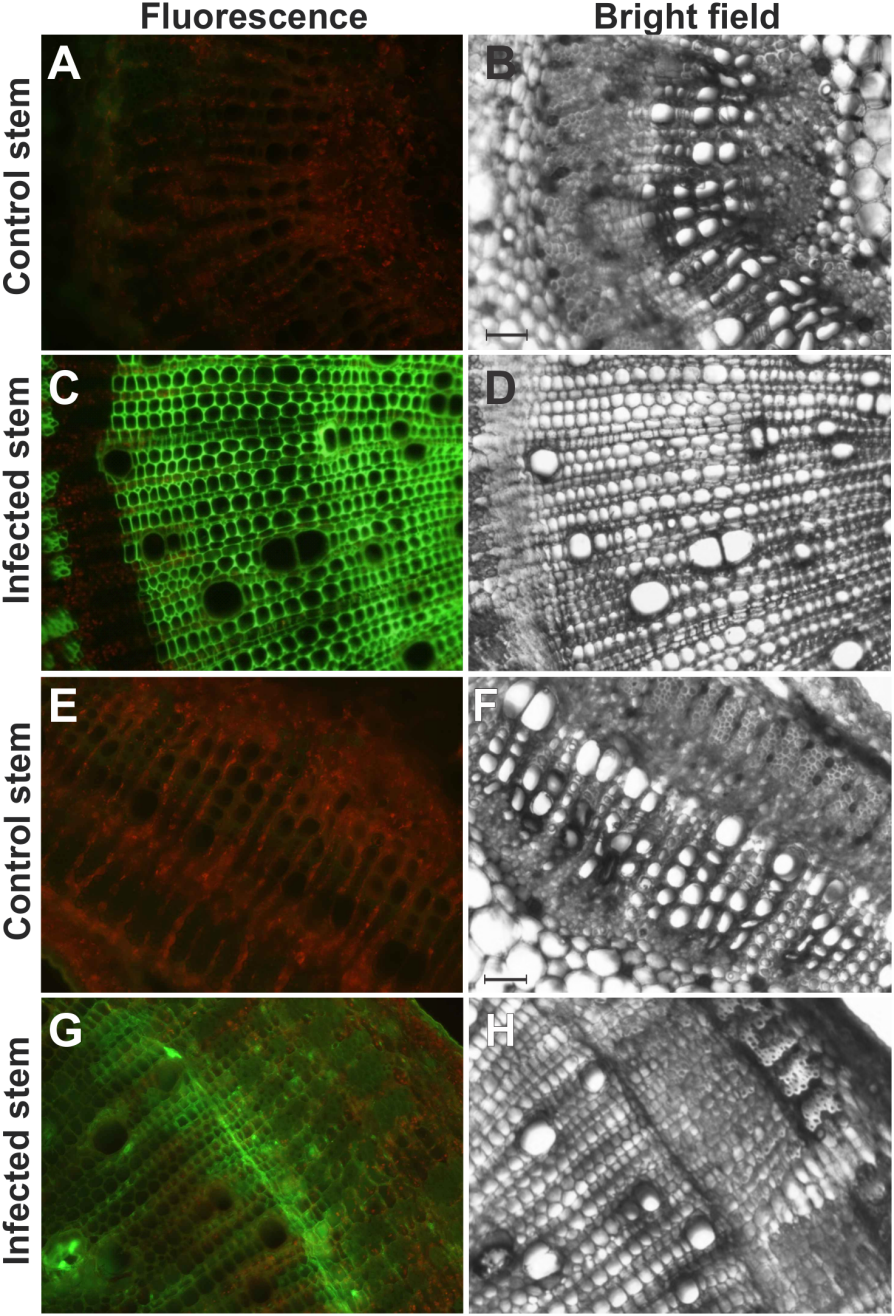
Detection of RNS and RSNO in Jute stem. DHR 123 and Alexa fluor 488 Hg-link phenylmercury were used for detecting RNS and RSNO in control and infected jute stem cross sections respectively. Images are control (A) and infected (C) jute stem cross sections showing the bright green fluorescence corresponded to the detection of RNS. (B) and (D) are the corresponding bright fields of (A) and (C) respectively. The red colour corresponds to the autofluorescence. Detection of RSNO in control (E) and infected (G) stem cross sections showing the bright green fluorescence. (F) and (H) are corresponding bright fields respectively. Figures are representative of at least six independent experiments. Bar = 250 µm.

When the infected stem sections were stained with DHR 123, RNS specific bright green fluorescence was observed in infected stem tissues (Figure 5C). There was no RNS specific fluorescence in control plant (Figure 5A). Dihydrorhodamine 123 has been reported to react with reactive oxygen species as well as reactive nitrogen species. In order to determine the specificity of DHR 123, infected tissue sections were further tested for ROS and RSNO detection using fluorescent probes. Surprisingly, there was no ROS specific fluorescence in infected tissues as evidenced with DCFDA (Figure S2), and subsequent presence of RSNO, as evidenced by bright green fluorescence in presence of Alexa fluor488, indicated the induction of RNS occurred following *M. phaseolina* invasion.

The potent Hg-link phenylmercury compounds form stable thiolates with free sulfhydryls, but can also react with other thiol moieties, including nitrosylated thiols. Cellular thiols could easily react with nitric oxide to produce nitrosylated thiols or RSNO, so the RSNO content in control and infected stems of *C. capsularis* were compared using epifluorescence and phase contrast microscopy. A representative of infected stem sections of susceptible (JRC 412) variety has been shown here (Figure 5G). Interestingly, nitrosylated thiols were also concentrated in the interface of phloem and xylem region. There was practically no significant fluorescence intensity in control stem (Figure 5E).

**Figure 6:**
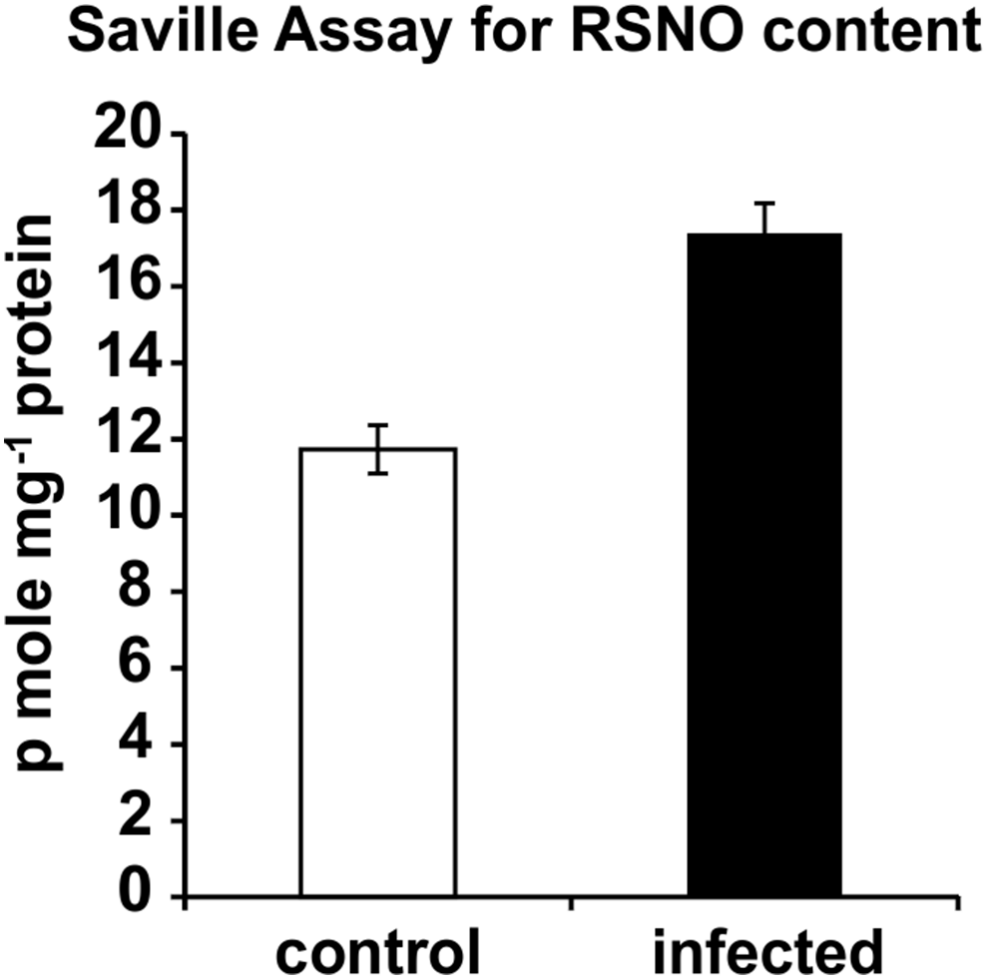
Quantitative measurement of RSNO in control *and M. phaseolina* infected jute leaf extract. RSNO contents were measured in crude leaf extracts according to the method described in experimental procedures. Results are expressed as mean ± SD, for n = 3 experiments. P≤0.01, using one-way ANOVA.

Nitrosothiol formation was further confirmed by Saville assay which is specific for quantitation of nitrosothiols in terms of p mole nitrite mg^−1^ protein (Figure 6). Nitrosothiol content was significantly higher in infected stem of susceptible (JRC 412) variety (17.4±0.78 p mole mg^−1^ protein) than in the control (11.73±0.64 p mole mg^−1^ protein).

### Induction of NO in *C. capsularis* requires 8 hour post inoculation time with *M. phaseolina*

During the infection of *M. phaseolina* on *C. capsularis*, the production of NO was followed after 20 days of post inoculation when the disease had already been established. To determine the minimum time required for NO production in *C. capsularis,* single leaf infection study was followed. The presence of NO in leaf tissues of the control and inoculated susceptible plant was analyzed using cell permeable NO specific probe DAF-FM DA. When the cultivar JRC 412 (susceptible) was infected with *M. phaseolina*, differential NO generation pattern was detected in different hours of post inoculation (Figure S3, S4, S5). Infected leaf sections showed high fluorescence in xylem, phloem, epidermis, spongy and palisade mesophyll also. This signal did not correspond to auto fluorescence, as it was not observed in the absence of DAF-FM. The fluorescence intensity was much higher in the 8 hour post inoculation in the susceptible variety (Figure S5).

### Induction of NO in *C. capsularis* leaves in the presence of fungal secretome

Plant cell wall represents the first barrier to an invading pathogen. Invasive plant pathogens produce enzymes like cellulase or hemicellulase to disrupt cellulose or hemicellulose component of the host cell wall which leads to stress or change in the cell wall associated pattern. In consequence of it, host plant may recognize its own damaged –self through damage associated molecular pattern molecules (DAMPs) and the stress or change is perceived by a sensor so that the plant responds to the change in a defensive manner.

**Figure 7:**
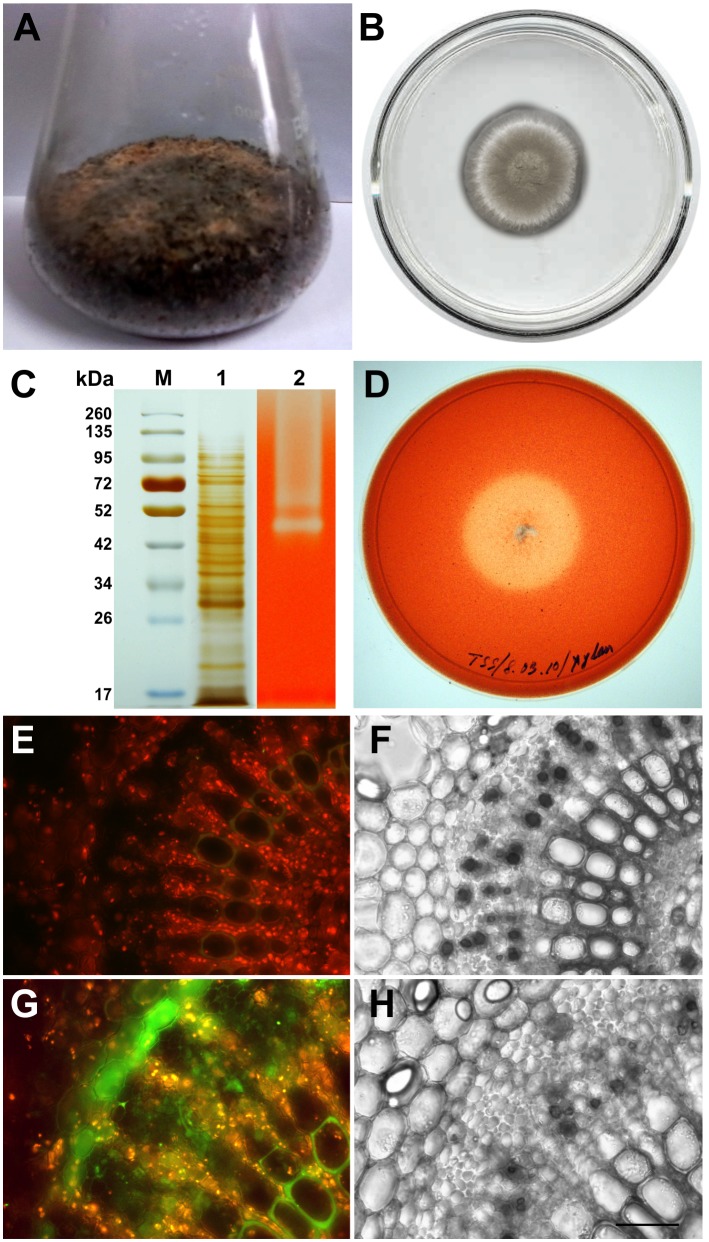
Secretome analysis of *M. phaseolina* and xylanase induced NO production in jute leaf discs. *M. phaseolina* were grown on wheat bran under Solid State Fermentation (SSF) (A). (B) represents *M. phaseolina* culture grown on birch wood xylan containing agar plate. (C) represents silver stained gel *M. phaseolina* secretome. M corresponds to molecular weight markers and lane 1 represents the fungal secretome. Lane 2 represents zymogram analysis of xylanase. Achromatic white bands represent strong xylanase activity of the fungal secretome. (D) represents Congo Red stained plate of *M. phaseolina* culture grown on birch wood xylan containing agar plate (B) showing light yellow coloured hallow zone for xylanase activity. Panel (E) and (G) represent control and xylanase treated leaf discs cross sections respectively. Xylanase induced NO production was detected with NO specific fluorophore DAF FM-DA showing the bright green fluorescence corresponded to the presence of NO. The orange yellow colour corresponds to the autofluorescence. (F) and (H) are the corresponding bright fields of (E) and (G) respectively. Figures are representative of at least six independent experiments. Bar = 400 µm.

To mimic the situation, *M. phaseolina* was grown on wheat bran for 7 days in a solid state fermentation (SSF) as well as birch wood xylan agar (Figure 7A, B). Extracellular secretome was collected from the SSF after downstream processing. SDS-PAGE analysis of the fungal secretome also showed a large number of proteins (Figure 7C). Fungal secretome was further subjected to Zymogram analysis for xylanase activity (Figure 7C). *M. phaseolina* was also grown in birch wood xylan agar to determine its potential for xylanase production. Zymogram analysis as well as Congo red staining of the inoculated birch wood xylan agar plate revealed that the necrotrophic fungus *M. phaseolina* could produce highly active extracellular xylanase (Figure 7D). In fact, xylanase is known to play a vital role in presenting pathogen associated molecular pattern (PAMP) which is evident in different model systems [20], [21]. In tomato cells, NO has been shown to be involved in the induction of phosphatidic acid (PA) production in response to the PAMP xylanase [22]. Interestingly, when leaf discs were submerged in the crude xylanase, NO generation was observed in the incubated tissue sections after 8 hour post incubation (Figure 7G). Although the role of xylanase as a PAMP has, indeed, been reported previously in other pathosystems, the response observed in this system may be due to the xylanase functioning as a PAMP, but an effect from other/s protein/proteins can’t be discarded as the leaf discs are exposed to a complex mixture of proteins.

### NO production by *M. phaseolina*

**Figure 8:**
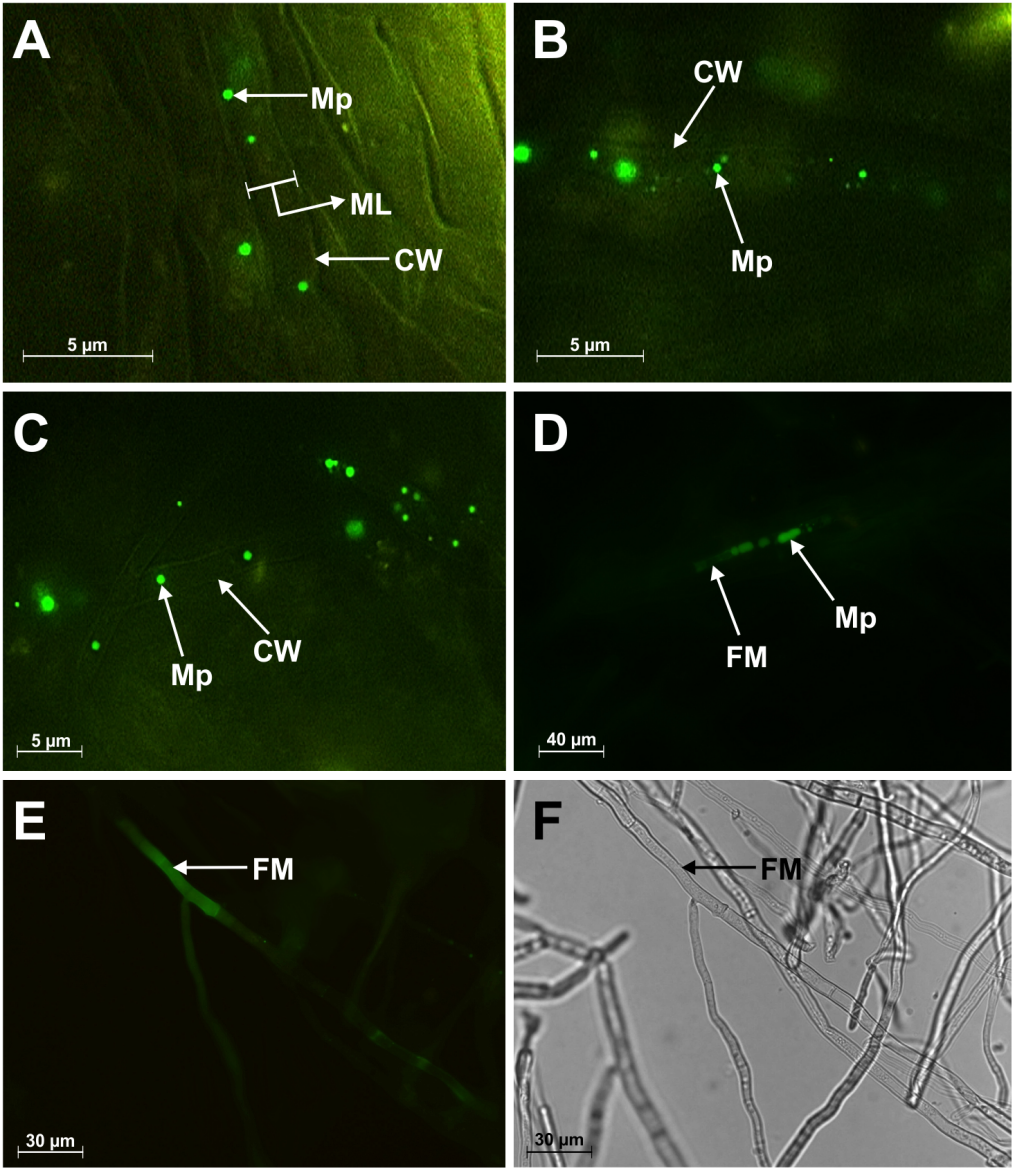
Detection of NO in the micro particle present in the mycelia of *M. phaseolina*. Micro particle, Mp; mycelial lumen, ML; fungal mycelium, FM; cell wall of fungal mycelium, CW. Individual or separate micro particles shown in panel (A), (B) and (C). Panel (D) and (E) represent diffusion of NO within the mycelia as shown by bright green fluorescence in presence of DAF-FM. Panel (F) is the corresponding bright field of panel (E).

In the present study it was evidenced that *M. phaseolina* infected tissue sections contained lot of NO. In recent studies it has been shown that necrotrophic fungi *B. cinera* could produce NO under *in vitro* conditions [23]. It is possible that initially NO synthesis came from the host and next, to a large extent, from the pathogen. To monitor the production of NO, *M. phaseolina* was grown in liquid culture and fungal mycelia was incubated with nitric oxide specific fluorescent probe DAF-FM. Interestingly, strong NO specific bright green fluorescence was observed within the mycelia and in the surrounding culture media up to 24 hour after the initial time of incubation. High resolution fluorescence microscopy revealed some micro particle like structure (Figure 8) generating NO continuously within the fungal mycelia. Control experiments with the NO scavenger cPTIO, did not show any NO specific fluorescence. This provided evidence of the specificity of the signal detected in the experiments conducted to investigate the fungal production of NO (Figure S6 Panel A). Due to its very short life, NO is readily oxidized to nitrite and nitrate. So the nitrite content of the media was also determined using Griess assay. *M. phaseolina* could produce 4.22 µM nitrite ml^−1^ after 24 hours of incubation. The well-known nitric oxide synthase inhibitor L-NAME was also applied to *M. phaseolina* liquid culture media for 16 hour to find whether NO production was NOS dependent or not. Then similar fluorescence microscopic study was carried out using DAF-FM DA. Surprisingly, NOS inhibitor could prevent the continuous NO productions in fungal mycelia as evidenced by fluorescence microscopy (Figure S6 Panel C). This experiment provided an indication for the existence of NOS like protein in *M. phaseolina*.

During this study the *M. phaseolina* genome has been sequenced. Interestingly, a Flavodoxin/Nitric Oxide Synthase protein with a calculated molecular weight of 69 kDa has been reported for *M. phaseolina*. Sequence homology analysis was conducted to find the conserveness of the NOS sequence reported in *M. phaseolina*. *M. phaseolina* NOS sequence showed conserved amino acid sequences if it is compared with the other reported NOS sequences.

### In silico analysis of NOS sequence of *M. phaseolina*

The 22 NOS sequences of various organisms starting from human to the bacteria, collected from NCBI database (Table 1) were aligned using MEGA 5 by the MUSCLE algorithm using default parameters which showed very few conserved amino acids among the sequences. Since the sequences of NOS proteins chosen belonged to species with very diverse evolutionary background as for e.g. bacteria, alga, fungi and mammals this might contribute to such a few number of exact matches of amino acids.

**Table 1:** List of the 22 NOS sequences of various organisms collected from NCBI database.

Sl No.	Name of the organism/microorganism	Accession number of the NOS sequence
1.	*Macrophomina phaseolina* MS6 Flavodoxin/nitric oxide synthase	gi|407927822|
2.	*Neofusicoccum parvum* UCRNP2 putative nitric oxide synthaseprotein	gi|485924258|
3.	*Colletotrichum gloeosporioides* Nara gc5 nitric oxide synthase	gi|429852284|
4.	*Glomerella graminicola* M1.001 nitric oxide synthase	gi|310800806|
5.	*Aspergillus oryzae* RIB40 nitric oxide synthase	gi|317156281|
6.	*Physarum polycephalum* nitric oxide synthase form B	gi|126253866|
7.	*Homo sapiens* NOS2_HUMAN Nitric oxide synthase	sp|P35228|
8.	*Drosophila melanogaster* NOS_DROME Nitric oxide synthase	sp|Q27571|
9.	*Bacillus subtilis* (strain 168) NOSO_BACSU Nitric oxide synthaseoxygenase	sp|O34453|
10.	*Bombyx mori* NOS_BOMMO Nitric oxide synthase	sp |Q8T8C0|
11.	*Deinococcus radiodurans* NOSO_DEIRA Nitric oxide synthaseoxygenase	sp|Q9RR97|
12.	*Bos taurus* NOS3_BOVIN Nitric oxide synthase, endothelial	sp|P29473|
13.	*Homo sapiens* NOS3_HUMAN Nitric oxide synthase, endothelial	sp|P29474|
14.	*Rattus norvegicus* NOS2_RAT Nitric oxide synthase, inducible	sp|Q06518|
15.	*Mus musculus* NOS3_MOUSE Nitric oxide synthase, endothelial	sp|P70313|
16.	*Rattus norvegicus* NOS3_RAT Nitric oxide synthase, endothelial	sp|Q62600|
17.	*Mus musculus* NOS2_MOUSE Nitric oxide synthase, inducible	sp|P29477|
18.	*Staphylococcus aureus* NOSO_STAAU Nitric oxide synthaseoxygenase	sp|P0A004|
19.	*Mus musculus* NOS1_MOUSE Nitric oxide synthase, brain	sp|Q9Z0J4|
20.	*Homo sapiens* NOS1_HUMAN Nitric oxide synthase, brain	sp|P29475|
21.	*Cavia porcellus* NOS2_CAVPO Nitric oxide synthase, inducibleFlavodoxin/nitric oxide synthase	sp|O54705|
22.	*Ostreococcus tauri* Q00TT5_OSTTA Nitric oxide synthase (ISS)	tr|Q00TT5|

**Figure 9:**
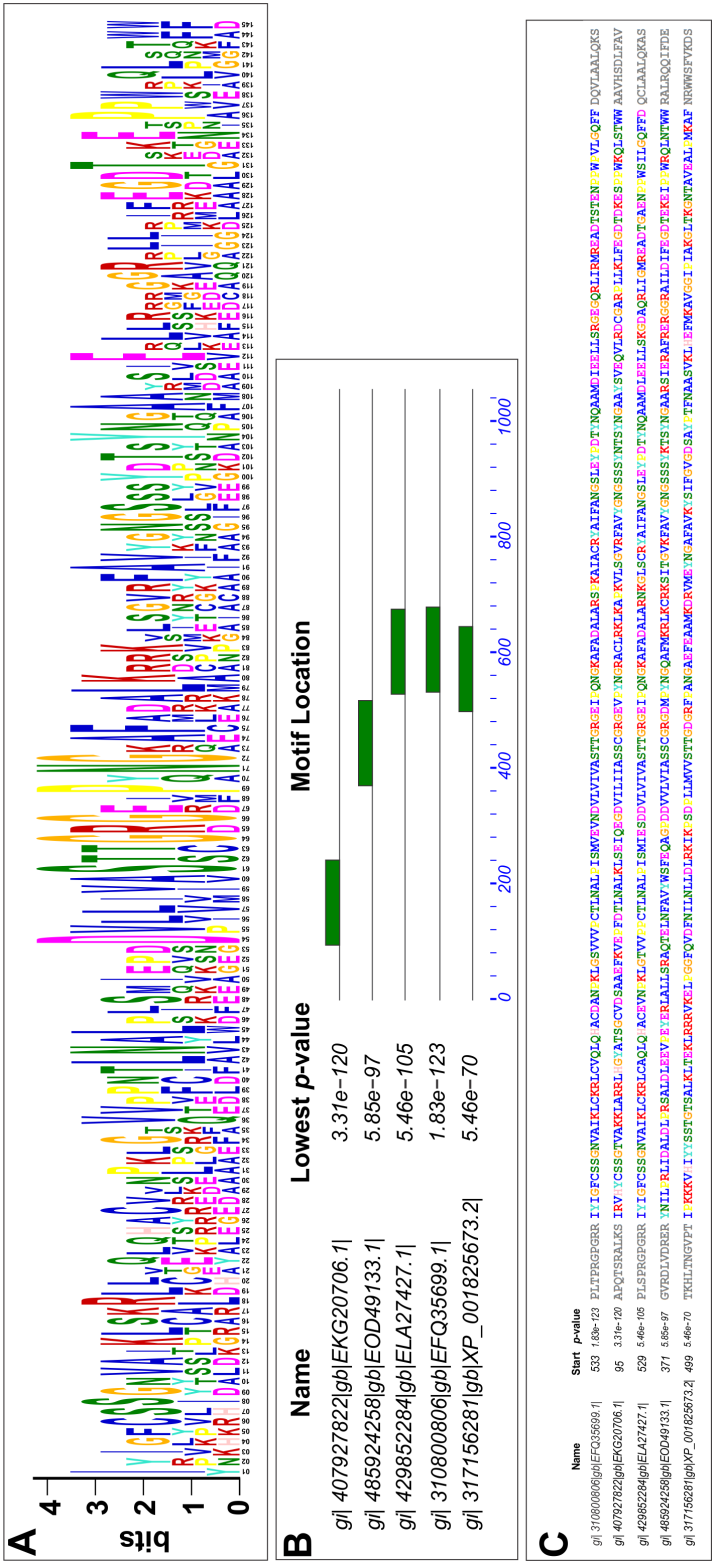
Motif enrichment analysis of the reductase domain of *M. phaseolina* MS6 with the four fungal NOS sequences by MEME. Enriched motif (A) found in 5 NOS sequences from the species *M. phaseolina*, *N. parvum*, *C. gloeosporioides*, *G. graminicola*, *A. oryzae* using MEME. (B) represents positions of the enriched motif in the five fungal NOS sequences. The motif is actually the Flavodoxin domain in these 5 necrotrophic pathogens. (C) represents the actual sequence of the enriched motif in the five fungal NOS sequences. The very low p-value denotes the high stringency in occurrence in each of the sequence.

**Figure 10:**
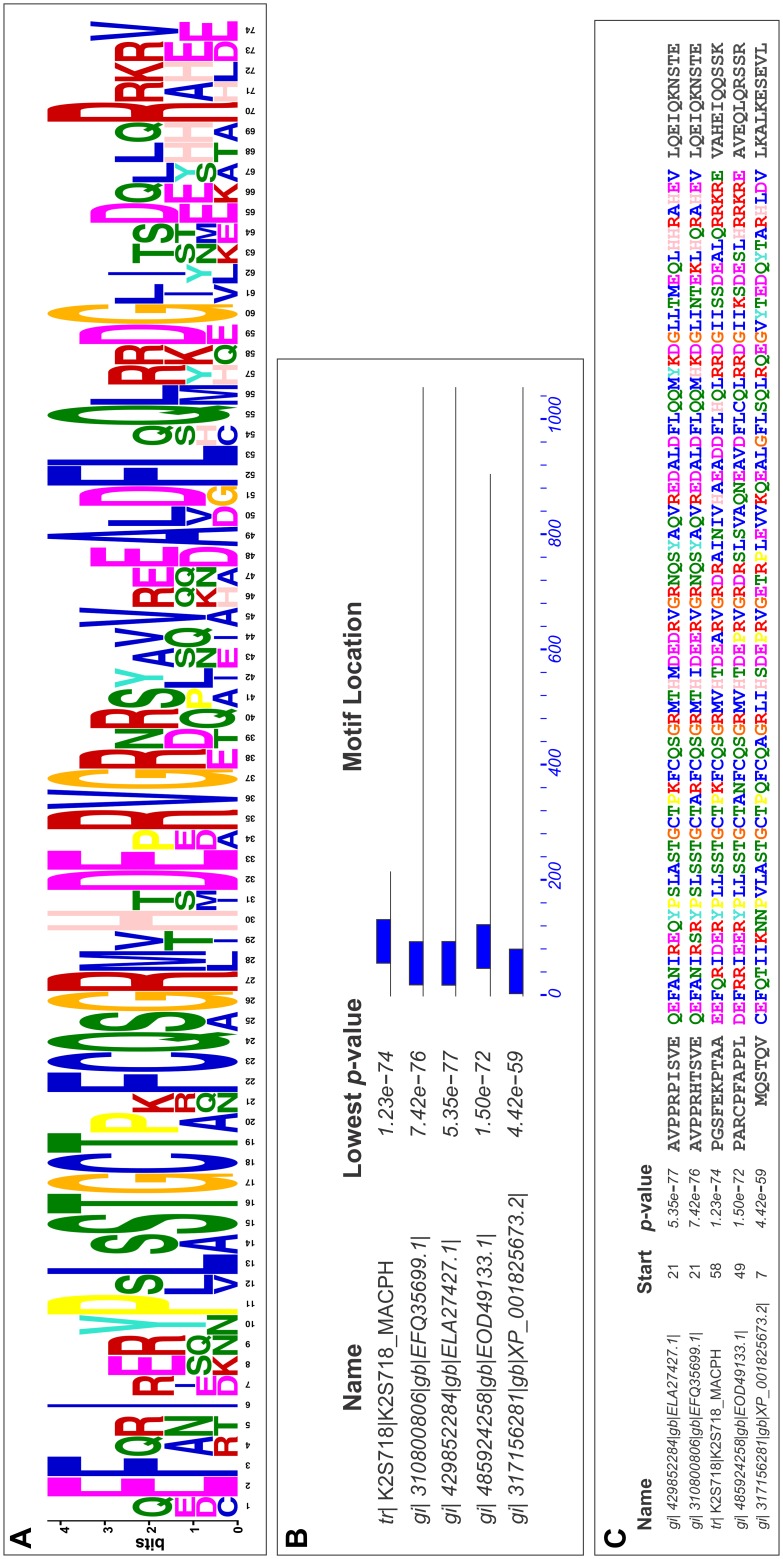
Motif enrichment analysis of the putative oxygenase domain of *M. phaseolina* MS6 with the four fungal NOS sequences by MEME. Enriched motif (A) found in 5 NOS sequences from the species *M. phaseolina*, *N. parvum*, *C. gloeosporioides*, *G. graminicola*, *A. oryzae* using MEME. (B) represents position of the enriched motif in the five fungal NOS sequences. The motif is actually the Oxygenase domain in these 5 necrotrophic pathogens. (C) represents the actual sequence of the enriched motif in the five fungal NOS sequences. The very low p-value denotes the high stringency in occurrence in each of the sequence.

Since very few conserved amino acids were found among all the selected NOS sequences, motif enrichment was carried out using the above-mentioned 22 NOS sequences. One motif consisting of 145 amino acids long (Figure 9A) was found to be enriched in five sequences out of the 22 sequences with very low p-values i.e. very high stringency (Figure 9B). Those five NOS sequences in which the enriched motif was found to be present were as follows: Flavodoxin/nitric oxide synthase of *Macrophomina phaseolina* MS6 (gi|407927822|gb|EKG20706.1), putative nitric oxide synthase protein *Neofusicoccum parvum* UCRNP2 (gi|485924258|gb|EOD49133.1), nitric oxide synthase *Colletotrichum gloeosporioides* Nara gc5 (gi|429852284|gb|ELA27427.1), nitric oxide synthase *Glomerella graminicola* M1.001 (gi|310800806|gb|EFQ35699.1) and nitric oxide synthase *Aspergillus oryzae* RIB40 (gi|317156281|ref|XP_001825673.2). Interestingly, seven amino acids of those sequences were highly conserved among them with enriched motif while the other amino acids were variable (Figure 9C). It was quite exciting to see that all the five species containing NOS sequences with enriched motif were necrotrophic pathogens. Apart from the flavodoxin/nitric oxide synthase of *M. phaseolina* MS6 (gi|407927822|gb|EKG20706.1), we have also considered its adjacent upstream ORF K2S718_MACPH. *M. phaseolina* MS6 K2S718_MACPH sequence was used to search for enriched motifs along with the four NOS sequences from the previously mentioned species. Interestingly, twenty seven amino acids of those sequences were highly conserved out of the seventy four amino acids in the enriched motif while the other amino acids were variable (Figure 10 A, B, C). The enriched motif was found to reside in the oxygenase domain of the four NOS sequences (Figure 10B) apart from *M. phaseolina* MS6 K2S718_MACPH, which indicated that this uncharacterized protein of *M. phaseolina* MS6 K2S718_MACPH could actually possess the oxygenase domain of its probable NOS protein.

**Figure 11:**
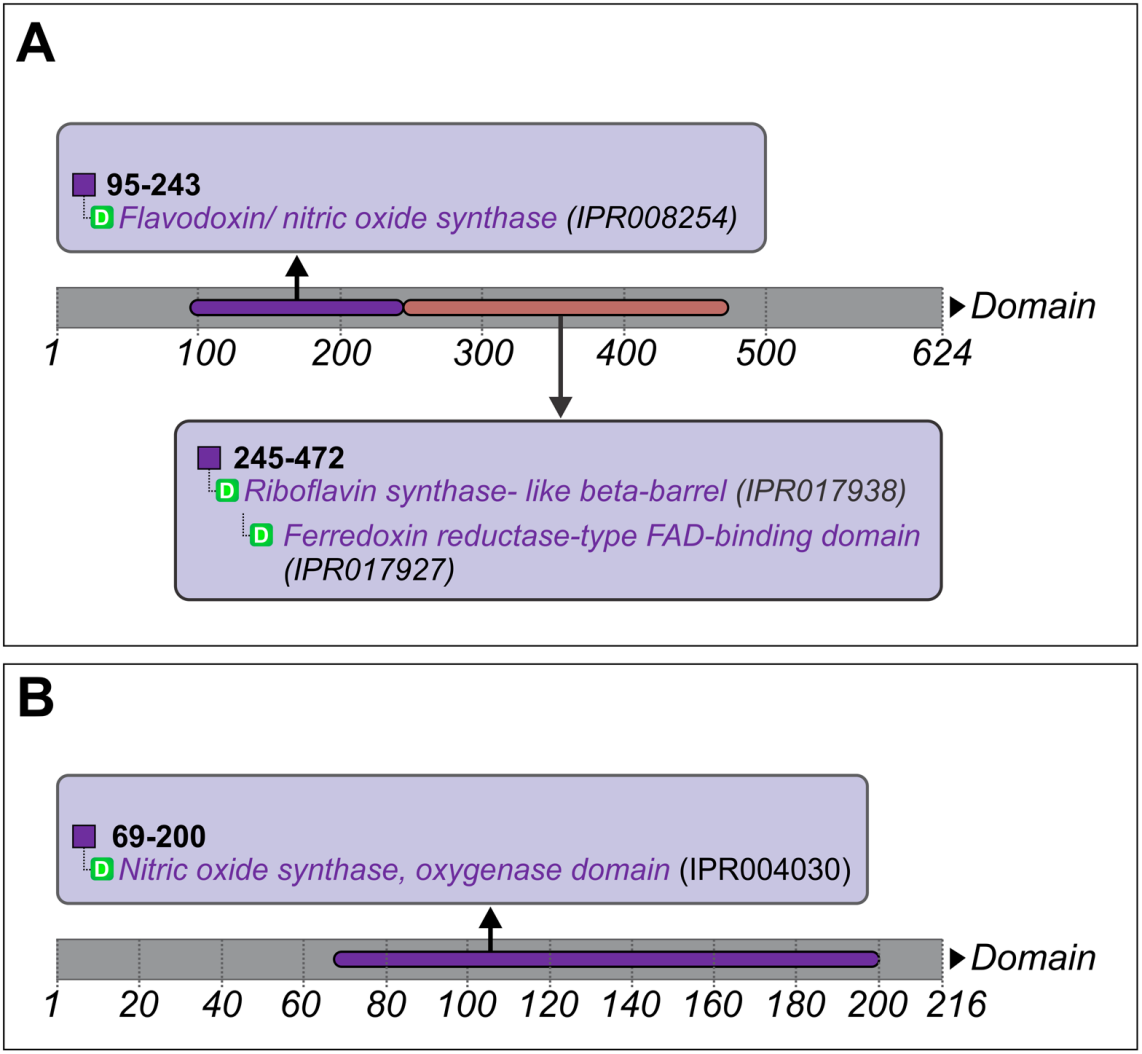
Flavodoxin/nitric oxide synthase and Oxygenase domain of Nitric oxide synthase of *M. phaseolina* MS6. Panel A represents the predicted domain structure of Flavodoxin/nitric oxide synthase which was found to be present in the reported amino acid sequence of *M. phaseolina* MS6. The Flavodoxin domain corresponds to amino acids 95 to 243. Panel B represents the predicted domain structure Oxygenase domain which was found to be present in the reported amino acid sequence of *M. phaseolina* MS6.

Further to find out the exact domain structure in which the enriched motifs of both *M. phaseolina* MS6 (gi|407927822|gb|EKG20706.1) and K2S718_MACPH resided, each of the five sequences was analyzed by INTERPRO to predict the probable domain structure in the amino acid sequence. It was discovered that *M. phaseolina* MS6 (gi|407927822|gb|EKG20706.1) did not possess an oxygenase domain as found in the other four sequences. Flavodoxin/nitric oxide synthase of *M. phaseolina* MS6 actually had two well defined motifs (Figure 11A), the Flavodoxin domain and the FAD binding domain which were present in the other four sequences too. On the other hand, *M. phaseolina* MS6 K2S718_MACPH possessed a predicted oxygenase domain structure which was also found in the four other NOS sequences (Figure 11B).

Next the two domains i.e. the flavodoxin domain and the oxygenase domain which were found to be enriched in all the five sequences, were again aligned by MEGA 5 (Figure S7 and S8). The oxygenase domain in *M. phaseolina* MS6 K2S718_MACPH gave an exact match of forty four amino acids with the oxygenase domains of the four other necrotrophic fungi NOS sequences. The flavodoxin/nitric oxide synthase of *M. phaseolina* MS6 gave an exact match of eighty seven amino acids with the flavodoxin domain and the FAD binding domain of the four other necrotrophic fungi NOS sequences.

**Figure 12:**
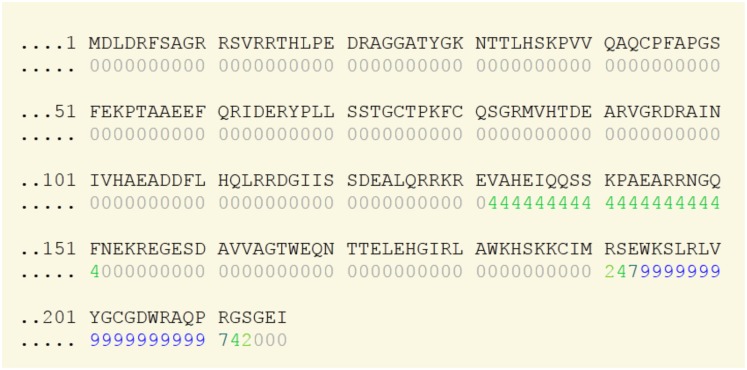
Putative CaM binding motif within the oxygenase domain of *M. phaseolina* MS6. The stretch of amino acids with a score of 9 represents probable CaM binding region.

Since it is well known that NOS proteins have a calmodulin (CaM) binding site in their sequences, we were interested to find out probable CaM binding sites in each of our *M. phaseolina* MS6 domains i.e. oxygenase and flavodoxin/nitric oxide synthase. We used the online tool Calmodulin Target Database (http://calcium.uhnres.utoronto.ca/ctdb/ctdb/home.html) to search for probable CaM binding sites in the two above mentioned domains. The K2S718_MACPH which contains the oxygenase domain had a stretch of seventeen amino acids with a score of 9 (Figure 12) which is actually the highest score allotted by the tool indicating very strong affinity for CaM binding. The EKG20706.1 which contains the flavodoxin/nitric oxide synthase domain did not possess such a stretch of amino acids with a high score indicating that CaM binding sites may not be present in this domain.

### Status of the redox active enzymes in *C. capsularis*: *M. phaseolina* interaction

**Figure 13:**
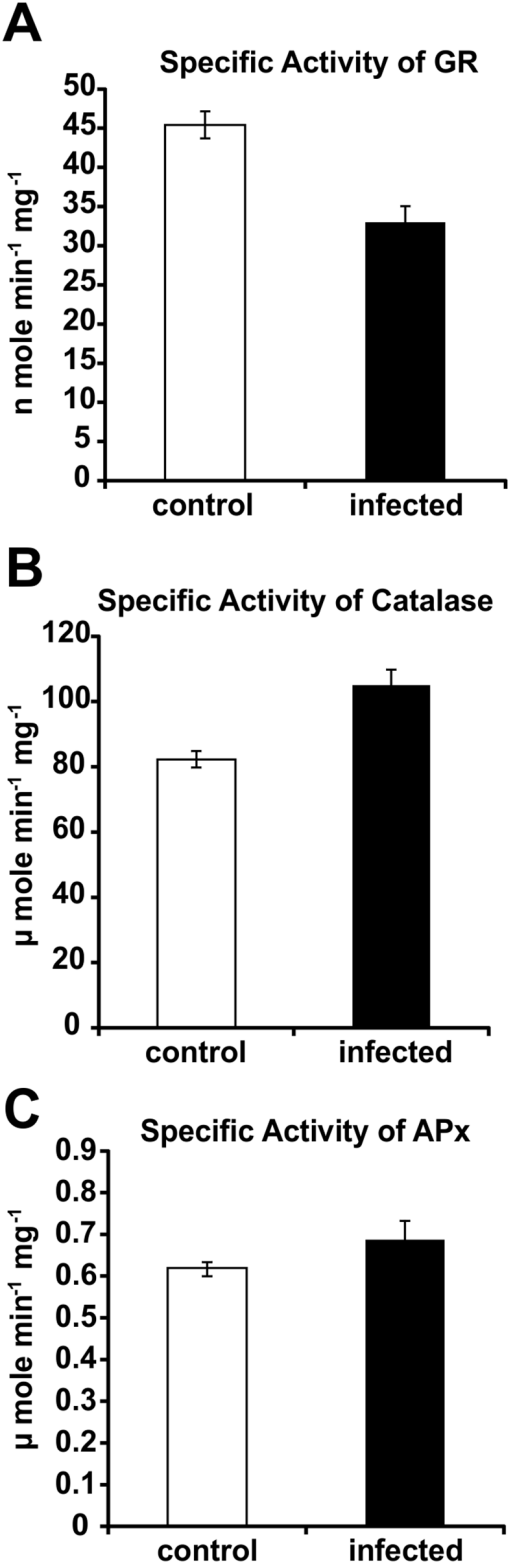
Determination of GR (A), Catalase (B) and APx (C) in control and *M. phaseolina* infected leaf extract. Enzymes were assayed in crude leaf extracts according to the method described in Materials and Methods. Results are expressed as mean ± SD, for n = 3 experiments. *p*≤0.01, using one-way ANOVA.

There are some reports in which enhanced reactive oxygen species (ROS) generation was found to accompany an infection caused by necrotrophs [24], [25]. In the context of early defense response events, the potential interplay of NO and ROS seems to be of special interest. We observed NO production during progression of the charcoal rot disease in *C. capsularis* caused by *M. phaseolina*. However, we did not observe any ROS specific fluorescence when the symptoms were visible in the infected tissues. It still remains to be determined the status of redox active enzymes. Glutathione Reductase (GR) is an important enzyme in maintaining the reducing environment of the cell. GR activity was found to be decreased in the infected sample in comparison with the healthy control plant. Inhibition of GR under nitrosative stress has been reported previously [26]. Interestingly, Catalase, one of the important ROS detoxification enzymes was increased in the infected condition. Ascorbate Peroxidase activity was also slightly increased followed by infection with *M. phaseolina* which correlated well with the absence of ROS specific fluorescence in the late stage of infection (Figure 13).

## Discussion

This study showed for the first time that NO, RNS and RSNO were produced in compatible *C. capsularis* JRC 412: *M. phaseolina* interaction. *M. phaseolina* is a necrotrophic pathogen that kills host cells and feeds on the remains for their own growth. NO and ROS are well known signaling molecules in disease resistance to necrotrophic pathogens but their role is not fully understood. It has been reported that an early NO burst serves as a source of secondary wave like NO generation in pelargonium (*Pelargonium peltatum*) plants resistant to the necrotrophic *Botrytis cinerea,* which probably stimulated a HR.

In this study a strong NO burst was observed in susceptible *C. capsularis* JRC 412 after 20 days post inoculation when the disease symptoms were very much prominent in Jute plant. Susceptible variety of *C. capsularis* JRC 412 also showed NO generation at the site of infection within 4 hour of leaf inoculation with *M. phaseolina* which reached maximum after 8 hour post inoculation. The importance of NO in plant resistance has been reported against the following fungi: In *B. cinerea,* and *Sclerotinia sclerotionum etc*. NO acts as a signaling molecule to activate diverse plant defense systems. At a very low NO concentration, it can function as a signaling molecule. On the other hand, high concentrations of NO may facilitate fungal infection by triggering plant cell death. Presence of NO, RNS and RSNO in the diseased stem of *C. capsularis* infected by *M. phaseolina* supported the hypothesis. There was no ROS specific fluorescence observed under the experimental conditions in susceptible *C. capsularis* JRC 412: *M. phaseolina* interaction. However, strong H_2_O_2_ accumulation, weaker NO burst and a lack in/of the wave of induced generation have been reported in a susceptible response of pelargonium (*P. peltatum*) leaves to *B. cinerea.* The existence of multiple pathways involving high NO, RNS and low NO, H_2_O_2_ could be possible for the susceptibility to necrotrophic pathogens. It is clearly evident from this study that induction of NO requires the presence of PAMP like xylanase. Extracted fungal secretome/crude xylanase treated leaf disc showed induction of NO at the same post inoculation time, like the susceptible leaf infected by *M. phaseolina.*

*M. phaseolina* produces NO both *in vitro* and *in planta*. This study showed for the first time, that NO generation in *M. phaseolina* is NOS dependent which was inhibited by L-NAME, a mammalian NOS inhibitor. NOSs are broadly distributed in the biological kingdom, starting from prokaryotes to eukaryotes. Proteins similar to mammalian NOS have been identified in *Deinococcus radiodurans* [27], *Bacillus subtilis* [28], *Staphylococcus aureus* [29] and in slime mold *Physarum polycephalum* [30], [31]. Fungi and plants do not contain NOS like sequences in their genomes, except for some fungal species from the genus *Aspergillus* (*A. flavus, A. oryzae* and *A. niger*) and *Glomerella graminicola.* An extensive genome analysis failed to reveal any NOS like sequence in *B. cinerea*, the most well studied necrotrophic pathogen in terms of molecular mechanism of disease pathology. Interestingly, *M. phaseolina* possess NOS like sequence. Not only that, bioinformatics analysis of *M. phaseolina* NOS like sequence revealed some very exciting information regarding the necrotrophic pathogens. Multiple alignments of NOS sequences using MEGA 5 followed by Motif enrichment using MEME generated two motifs, one in the oxygenase domain and the other in the flavodoxin/nitric oxide synthase domain which were present in separate ORFs. Surprisingly, these motifs are conserved among the five necrotophic pathogens. Further, the domain analysis of those five sequences showed diversity among them through evolution but being necrotrophic pathogens by nature, they shared homology among their oxygenase domains as well as flavodoxin domains.

Early studies of *D. radiodurans* (drNOS) demonstrated that NO synthesis could be supported by a surrogate mammalian reductase domain supplied in trans. Flavin-containing reductases from *B. subtilis* were tested for their ability to reduce bsNOS. Thus, it appears that many different types of reductase proteins can support NO synthesis and that, in the case of *B. subtilis*, a dedicated reductase may not be required at all *in vitro*. There is one exception to the stand-alone bacterial NOS proteins, and that is a NOS sequence found in the genome of a gram-negative bacterium, *Sorangium cellulosum*. The *S. cellulosum* NOS (scNOS) contains a covalently attached reductase module. However, the domain organization and cofactor complement in scNOS is unlike that found in other NOSs [48]. Thus it appears that the NO production in *M. phaseolina* can be explained even if the domains are present in separate ORFs. This is the characteristic feature of NOS that two separate domains can reconstitute the holoenzyme if the substrates and cofactors are available.

*In vivo* cell imaging with DAF-FM DA showed randomly moving microparticles producing NO in a continuous fashion which is novel information regarding NO generating machinery in necrotrophic pathogen (data not shown). It seems like NOS like protein resides within the microparticles. The capacity of the fungus to produce NO that diffuses outside the fungal cells could have important physiological implications because NO is a potent plant signaling molecule. Production of NO by the necrotrophic pathogen is always advantageous in terms of disease progression. *M. phaseolina* could enhance the infection of plant cells through its own production of NO.

According to Delledonne et al [2], NO and H_2_O_2_ may function synergistically, inducing a hypersensitive cell death in incompatible interaction. Genetic and pharmacological approaches showed that reduced endogenous NO levels lead to increased levels of ROS. NO can react very quickly with superoxide to form peroxynitrite and thus decrease the amount of endogenous ROS. The amount of ROS availability also depends on redox active enzymes. In this study, the level of catalase has been found to increase in infected plants. APx activity was also found to change with a little increase in *C. capsularis*. High concentrations of NO and activity of the redox active enzymes could justify the absence of ROS generation in the infected plant. GR is an important enzyme for maintaining the reducing environment of the cell. Previously it was shown that inhibition of GR activity was due to protein tyrosine nitration under nitrosative stress [26]. In the present study inhibition of GR activity in the infected tissues compared to the control may be due to protein tyrosine nitration under diseased condition.

All these observations presented in this study introduce additional considerations into the analysis of the interactions in which *M. phaseolina* participates and highlight the complexity of the interplay between *M. phaseolina* and the plant *C. capsularis* during the establishment and progress of the fungal necrotroph-host plant interaction.

## Materials and Methods

### Chemicals

All the reagents are of highest purity and purchased from Sigma Chemical Co (St Louis, MO, USA) unless otherwise stated.

### Plant material used

Previous study on screening for stem rot tolerant jute accessions carried out at three different locations (CRIJAF and Budbud in West Bengal; and Sorbhog in Assam) revealed the field tolerance of nine accessions of *C. capsularis* [19]. Among those, a cultivated variety, JRC 412 showed susceptibility. In the present study, susceptible variety JRC 412 was used for all the experiments.

### Initial source of fungal inoculum and preparation of fungal culture

A virulent isolate of *M. phaseolina* (strain R9) was collected from Sorbhog, Assam, which is assumed to be one of the hot spot locations with respect to stem rot disease of jute. A pure mycelial culture generated through single sclerotia of this isolate, maintained in Potato Dextrose Agar (PDA) media at 28°C, served as the initial source of inoculum and used in challenge inoculation studies. For the mass culture, the pathogen was grown in Potato Dextrose Broth (PDB) and incubated at 28°C for 72 hrs.

### Inoculation methods used for *Macrophomina* infectivity study

#### Leaf inoculation

*A. Excised leaf inoculation under moist cotton condition.* The fully expanded fourth leaf from the top of one month old plants of JRC412 was excised and placed separately in sterile glass plates (35-mm diameter). At the basal portion of the leaves, surrounding the petioles, moist cotton was wrapped for maintenance of the leaf turgidity. A wound was created at the tip of the leaves. A piece of mycelial bed of Macrophomina (strain R9) from 48 hr grown culture in Potato dextrose Broth (PDB) was taken, excess PDB was removed by washing it in sterile water and placed separately over the wounded tip of the leaves of both the accessions. Plates were incubated in a growth chamber at 28°C with combined fluorescent and incandescent lights (145 to 290 Em–2s–1 intensity) on 12 hr photoperiod for 2 days. The progression of infection was measured by the length of the necrotic lesion.

*B. Leaf disc inoculation.* The fully expanded fourth leaf from the top of one month old plants of JRC412 was excised; a disc of 3 mm in diameter was cut and placed separately on sterile glass plate (35-mm diameter). A piece of mycelial bed of *Macrophomina* (strain R9) from 48 hr grown culture in Potato dextrose Broth (PDB) was taken and excess PDB was removed by washing it in sterile water. The excised leaf discs of both the accessions, placed in sterile glass plates, were separately inoculated with the mycelial bed. Such plates were then incubated in a growth chamber at 28°C with combined fluorescent and incandescent lights (145 to 290 Em–2s–1 intensity) on 12 hr photoperiod for 2 days.

*C. Stem inoculation.* Stems of 21 day old seedlings of JRC 412 were inoculated with pieces of infected toothpicks. The tips (1.0 to 1.5 cm long) of 50 wooden toothpicks were autoclaved for 20 min in 250 ml distilled water, removed, blotted, re-autoclaved in additional water to remove inhibitory substances. Toothpick pieces were then cooled in sterile Petri plates, transferred individually to margins of colonies of *M. phaseolina* maintained on PDA and incubated for 24 h in a growth chamber at 28°C with combined fluorescent and incandescent lights (145 to 290 Em–2s–1 intensity) on a 12 h photoperiod. An insertion was created at the side of the stem using a sterile razor and a single infested toothpick piece containing fungal propagule was inserted into the stem at 45° angle. The inserted region was sealed with para-film to prevent desiccation. The non-infected sterile toothpicks pieces incubated in sterile PDA media were also inserted in the similar way into the plants of JRC 412 and such plants served as the control plants. For establishment of the infection, inoculated plants were kept in a growth chamber at 37°C with 90% relative humidity.

### Detection of NO and RNS by fluorescence microscopy

NO was detected in jute leaf and stem cross sections that were incubated for 1 h at 25°C, in darkness, with 10 µM 4-Amino-5-Methylamino-2′,7′-Difluorofluorescein Diacetate (DAF-FM DA) prepared in 10 mM Tris–HCl (pH 7.4) [32]. Background staining, routinely negligible, was controlled with unstained sections. As control, sections were pre-incubated for 30 min at 25°C with 200 µM cPTIO, an ·NO scavenger. For Reactive Nitrogen Species, the samples were incubated with 10 µM DHR 123. After incubation, samples were washed twice in the same buffer for 15 min each. Then the sections were examined by Olympus BX51 fluorescence microscope attached with Olympus *CoolSNAP* cf color/OL camera using appropriate filter. Light intensity and exposure times were kept constant for a given set of experiment and collection modalities for DAF-FM DA green fluorescence (excitation 495 nm; emission 515 nm), DHR 123 green fluorescence (excitation 488 and emission 525–550 nm) and chlorophyll autofluorescence (chlorophyll a and b, excitation 429 and 450 nm, respectively; emission 650 and 670 nm, respectively) as orange.

### Detection of RSNO by fluorescence microscopy

RSNOs were detected using the fluorescent reagent Alexa fluor 488 Hg-link phenylmercury [33]. Tissue sections were incubated with 10 µM Alexa fluor 488 Hg-link phenylmercury (Molecular Probes, cat. no H30462) at 25°C for 2 h, in darkness, and then were washed three times in 10 mM Tris–HCl buffer, pH 7.4, for 15 min each. After washing three times in the previous buffer, then the sections were examined by Olympus BX51 fluorescence microscope attached with Olympus *CoolSNAP* cf color/OL camera using appropriate filter. Light intensity and exposure times were kept constant for a given set of experiment and collection modalities for Alexa fluor 488 green fluorescence (excitation 495 nm; emission 519 nm).

### Detection of Superoxide Radical and Reactive Oxygen Species by fluorescence microscopy

For superoxide radicals, the samples were incubated at 37°C for 30 min with 10 µM dihydroethidium (DHE), as described by Rodriguez- Serrano et al. [34] and for reactive oxygen species, samples were incubated with 10 µM DCF-DA) [35]. For positive control samples were incubated with 10 µM H_2_O_2_ for 30 min. After incubation, samples were washed twice in the 10 mM Tris–HCl buffer, pH 7.4 for 15 min each. Then the sections were examined by Olympus BX51 fluorescence microscope attached with Olympus *CoolSNAP* cf color/OL camera using appropriate filter. Light intensity and exposure times were kept constant for a given set of experiment and collection modalities for DHE green fluorescence (excitation 488 nm; emission 520), DCF-DA green fluorescence (excitation at 485 nm, emission at 530 nm) and chlorophyll autofluorescence (chlorophyll a and b, excitation 429 and 450 nm, respectively; emission 650 and 670 nm, respectively) as orange.

### Qualitative Detection of Xylanase activity of *M. phaseolina* by plate assay

Xylanolytic activity was qualitatively identified with the help of Congo red dye [36]. The *M. phaseolina* colony was grown on Xylan Agar media plate. The colony was then flooded with 0.1% aqueous Congo red dye for at least 1 h followed by destaining with 1 M NaCl. The plate was examined for the appearance of whitish yellow zone of hydrolysis around the colonies.

### Production of extracellular xylanase by Solid State Fermentation

The *M. phaseolina* strain was subcultured on potato dextrose agar (PDA). For the production of xylanase, sclerotia were collected from PDA slants in Mandel mineral salt solution [37] and spread over the solid bed (10^4^–10^6^ sclerotia per 5.0 g of wheat bran) uniformly maintaining the water activity of the solid bed below 0.6. Organisms were then allowed to grow at 30°C for 7 days. Enzyme was extracted from the wheat bran bed by agitating it in 50 mM sodium phophate buffer (pH-7) for 1 h in a shaker with 150 rev min^−1^. Supernatant of the agitated mixture was collected for the zymogram analysis after centrifugation at 3000 g for 30 min.

### Zymogram analysis

The zymogram analysis of Xylanase from SSF culture was performed according to Tseng et al. [38]. 10 µg crude enzyme samples were mixed with the same volume of loading buffer and boiled at 100°C for 1 min. After the separation of the enzyme samples by SDS-PAGE containing 2% birchwood xylan, the gel was divided into two parts. One part, containing the samples and molecular marker (Fermentas, USA), was stained using standard silver stain procedure. The other part of the gel was washed with 50 mM phosphate buffer with 25% isopropanol and kept in same solution for 1 hr at room temperature. After that the gel was again washed with 50 mM phosphate buffer pH −7.0 without isopropanol and kept with the same buffer for 30 min at room temperature. After that the gel was incubated at 37°C for 10 min. Finally the gel was stained with 0.1% Congo red solution followed by destaining with 1 M NaCl solution until pale-red hydrolysis zones appeared against a red background. The molecular weights of the isoforms were determined by correlating it with identical SDS –PAGE profile of protein molecular weight standard.

### Spectrophotometric detection of Nitrite using Griess Assay

One means to investigate nitric oxide formation is to measure nitrite (NO_2_^–^), which is one of two primary, stable and nonvolatile breakdown products of NO. This assay relies on a diazotization reaction that was originally described by Griess [39]. The Griess Reagent System is based on the chemical reaction between nitrite, sulfanilamide and *N*-1-napthylethylenediamine dihydrochloride (NED) under acidic (phosphoric acid) conditions. Sulfanilamide and NED compete for nitrite in the Griess reaction; thus to achieve greater sensitivity, the two components were added sequentially. Firstly, the Sulfanilamide Solution to the sample was added, incubated for 5–10 minutes, then the NED Solution was added and pink colour was formed. After that, the solution was measured at 540 nm. To ensure accurate NO_2_^–^ quantitation, a reference standard curve with the nitrite standard (0.1 M Sodium Nitrite in water) was prepared, using the same culture media or buffer used for experiment.

### Multiple sequence alignment

A total of 22 sequences from various species whose Nitric oxide synthase has been reported were selected for alignment using software MEGA 5 [40]. The algorithm used was MUSCLE [41] inside software MEGA under default parameters.

### Motif enrichment analysis

All the 22 sequences selected for multiple sequence alignment were submitted to the online tool MEME [42] to find out the enriched motifs among the sequences. Parameters used were like:

Occurrence of each motif: any no. of repetitions.
Width of each motif: varied from 6 to 150 amino acids
Number of motifs to return: 20
Using these parameters motifs were searched of varying lengths among the 22 sequences that were submitted to MEME.

### Predicting domains using INTERPRO

The FASTA sequence for Flavodoxin/nitric oxide synthase [*Macrophomina phaseolina* MS6] was submitted to INTERPRO [43] and using their exhaustive database searches, it was predicted to have two well characterized domains. The FASTA sequence of uncharacterized protein of *M. phaseolina* (strain MS6) (K2S718_MACPH) upon similar submission was predicted to have a single domain.

### Predicting Calmodulin binding sites using Calmodulin Target Database

The amino acid sequences of *M. phaseolina* MS6 K2S718_MACPH and EKG20706.1 were submitted to the Calmodulin Target Database (http://calcium.uhnres.utoronto.ca/ctdb/ctdb/sequence.html) to search for probable calmodulin binding sites. This tool allots a highest score of 9 to the amino acids it predicts to have highest affinity for calmodulin binding.

### Preparation of crude cell free extract

All operations were performed at 0–4°C. Jute leaves were ground to powder in a mortar with liquid nitrogen, and were suspended in 10 ml of 100 mM phosphate buffer (pH 7.4), containing 1 mM EDTA, 7% (W/V) PVPP, 15 mM DTT, 15 mM PMSF and centrifuged at 10000 r.p.m for 10 min to remove the cell debris. Then, the supernatants were used for the following experiments.

### Glutathione reductase (GR) activity assay

GR activity of crude cell free extract was determined by measuring the decrease in absorbance at 340 nm due to utilization of NADPH (extinction coefficient = 6.22 mM^−1 ^cm^−1^) [44]. The GR assay mixture contained 50 mM K-phosphate (pH 7.0), 1 mM EDTA, 1 mM GSSG, 0.15 mM NADPH and the enzyme in a total volume of 500 µl.

### Catalase activity assay

Catalase activity of crude cell free extract was determined by measuring the decrease in absorbance at 240 nm due to utilization of H_2_O_2_ (extinction coefficient = 43.6 M^−1 ^cm^−1^) [45]. The assay mixture contained 50 mM K-phosphate (pH 7.5), 1 mM EDTA, 10 mM H_2_O_2_ and the enzyme in a total volume of 500 µl.

### APX activity assay

Reaction mixture contained 50 mM potassium phosphate (pH 7.0), 0.1 mM H_2_O_2_ and 0.5 mM ascorbate. The H_2_O_2_-dependent oxidation of ascorbate was followed by monitoring the decrease in absorbance at 290 nm, assuming an absorption coefficient of 2.8 mM^–1 ^cm^–1^. One unit of APx was defined as the amount of enzyme that oxidized 1 µmol of ascorbate per min at 25°C [46].

### Biochemical measurement of S-Nitrosothiol Content with the Saville Assay

The S-nitrosothiol content was determined according to the Saville method [47]. For the analysis of the S-nitrosothiol content, three sets of solutions were prepared. Solution A consist 1% sulfanilamide dissolved in 0.5 M HCl, solution B contains 1% sulfanilamide and 0.2% HgCl_2_ in 0.5 M HCl and solution C was prepared by dissolving 0.02% N-(1-naphthyl)-ethylendiaminedihydrochloride in 0.5 M HCl. Protein samples of *C. capsularis* leaf extracts were prepared in 100 mM phosphate buffer (500 µl). Solution A (250 µl) was then added to one of the samples, and the same amount of solution B was added to the other. After 5 minute, when the formation of the diazonium salt was complete, 250 µl of solution C were added to each of the two samples. After 5 minute, color formation of the azo dye was complete and the absorbance at 540 nm was recorded with spectrophotometer. The absorbance at 540 nm of the reaction with solution A is for the presence of free nitrite in plant sample and the absorbance at the same wavelength with solution B is for the presence of free nitrite and S-nitrosothiol in plant sample. So the actual S-nitrosothiol content in plant sample is the deducted result of absorbance with solution B from absorbance with solution A. Finally the S-nitrosothiol content was determined in terms of nitrite from the standard curve of nitrite.

## Supporting Information

Figure S1 **Control experiment with NO scavenger cPTIO.** Representative images illustrate the scavenging of NO by cPTIO in *C. capsularis* stem and leaf. Stem section (A), leaf section (C) pre-incubated with the NO scavenger cPTIO (200 µM) (negative control). Corresponding bright fields are (B) and (D). The orange yellow color corresponds to the chlorophyll autofluorescence. Figures are representative of at least six independent experiments. Bar = 250 µm.
(TIF)

Figure S2 **Fluorescence microscopic images of control and infected jute stem sections for ROS detection.** (A) and (C) represent control and infected stem sections respectively. (B) and (D) are the corresponding bright field Images of (A) and (C) respectively. No ROS specific fluorescence was observed in all the sections. The red colour corresponds to the autofluorescence. Figures are representative of at least six independent experiments. Bar = 250 µm.
(TIF)

Figure S3 **Time dependent NO detection in control and infected jute leaf by fluorescence microscopy.** The cross sections of control jute stem showed no NO specific fluorescence (A). NO specific green fluorescence was detected in the cross sections of infected jute stem after 4 hour post inoculation (4 hpi)(C). (B) and (D) are the corresponding bright field images of (A) and (C) respectively. The red colour corresponds to the autofluorescence. Bar = 250 µm.
(TIF)

Figure S4 **Time dependent NO detection in control and infected jute leaf by fluorescence microscopy.** The cross sections of control jute stem showed no NO specific fluorescence (A). NO specific green fluorescence was detected in the cross sections of infected jute stem after 6 hour post inoculation (6 hpi)(C). (B) and (D) are the corresponding bright field images of (A) and (C) respectively. The red colour corresponds to the autofluorescence. Bar = 400 µm.
(TIF)

Figure S5 **Time dependent NO detection in control and infected jute leaf by fluorescence microscopy.** The cross sections of control jute stem showed no NO specific fluorescence (A). NO specific green fluorescence was detected in the cross sections of infected jute stem after 8 hour post inoculation (8 hpi) (C). (B) and (D) are the corresponding bright field images of (A) and (C) respectively. The red colour corresponds to the autofluorescence. Bar = 400 µm.
(TIF)

Figure S6 **Control experiment with NO scavenger cPTIO and L-NAME.** Representative images illustrate the effect of cPTIO and L-NAME in *M. phaseolina.* Fungal mycelia were pre incubated with the NO scavenger cPTIO (200 µM) Panel A and with L-NAME (Panel C). Absence of bright green fluorescence indicated the specificity of NO production. Corresponding bright fields are (B) and (D). Figures are representative of at least six independent experiments. Bar = 30 µm.
(TIF)

Figure S7 **Multiple sequence alignment of the reductase domain of** ***M. phaseolina*** **MS6 with the four fungal NOS sequences.** Five fungal NOS sequences of the following species *Macrophomina phaseolina*, *Neofusicoccum parvum*, *Colletotrichum gloeosporioides*, *Glomerella graminicola*, *Aspergillus oryzae* were used using MUSCLE algorithm in MEGA 5. The asterisks in the sequence positions denote the exact match of amino acids in all 5 sequences.
(TIF)

Figure S8 **Multiple sequence alignment of the putative oxygenase domain of** ***M. phaseolina*** **MS6 with the four fungal NOS sequences.** Five fungal NOS sequences of the following species *Macrophomina phaseolina*, *Neofusicoccum parvum*, *Colletotrichum gloeosporioides*, *Glomerella graminicola*, *Aspergillus oryzae* were used using MUSCLE algorithm in MEGA 5. The asterisks in the sequence positions denote the exact match of amino acids in all 5 sequences.
(TIF)

## References

[1] Jones JD, Dangl JL (2006) The plant immune system. Nature 444: 323–329. 17108957 10.1038/nature05286

[2] Delledonne M, Zeier J, Marocco A, Lamb C (2001) Signal interactions between nitric oxide and reactive oxygen intermediates in the plant hypersensitive disease resistance response. Proc Natl Acad Sci U S A 98: 13454–13459. 11606758 10.1073/pnas.231178298 PMC60892

[3] van Baarlen P, Staats M, van Kan JAL (2004) Induction of programmed cell death in lily by the fungal pathogen *Botrytis elliptica* . Mol Plant Pathol 5: 559–574. 20565630 10.1111/j.1364-3703.2004.00253.x

[4] Conrath U, Amoroso G, Kohle H, Sultemeyer DF (2004) Non-invasive online detection of nitric oxide from plants and some other organisms by mass spectrometry. Plant J 38: 1015–1022. 15165192 10.1111/j.1365-313X.2004.02096.x

[5] Floryszak-Wieczorek J, Arasimowicz M, Milczarek G, Jelen H, Jackowiak H (2007) Only an early nitric oxide burst and the following wave of secondary nitric oxide generation enhanced effective defence responses of pelargonium to a necrotrophic pathogen. New Phytologist 175: 718–730. 17688587 10.1111/j.1469-8137.2007.02142.x

[6] Wang J, Higgins VJ (2005) Nitric oxide has a regulatory effect in the germination of conidia of *Colletotrichum coccodes* . Fungal Genet Biol 42: 284–292. 15749048 10.1016/j.fgb.2004.12.006

[7] Prats E, Mur LA, Sanderson R, Carver TL (2005) Nitric oxide contributes both to papilla-based resistance and the hypersensitive response in barley attacked by *Blumeria graminis* f. sp. hordei. Mol Plant Pathol 6: 65–78. 20565639 10.1111/j.1364-3703.2004.00266.x

[8] Prats E, Carver TL, Mur LA (2008) Pathogen-derived nitric oxide influences formation of the appressorium infection structure in the phytopathogenic fungus *Blumeria graminis* . Res Microbiol 159: 476–480. 18554873 10.1016/j.resmic.2008.04.001

[9] Wyllie TD (1998) Soybean Diseases of the North Central Region. In: Wyllie TD, Scott DH, editors. Charcoal rot of soybean-current status: APS, St. Paul. 106–113.

[10] Islam MS, Haque MS, Islam MM, Emdad EM, Halim A, et al (2012) Tools to kill: genome of one of the most destructive plant pathogenic fungi *Macrophomina phaseolina* . BMC Genomics 13: 493. 22992219 10.1186/1471-2164-13-493 PMC3477038

[11] Su G, Suh SO, Schneider RW, Russin JS (2001) Host Specialization in the Charcoal Rot Fungus, *Macrophomina phaseolina* . Phytopathology 91: 120–126. 18944384 10.1094/PHYTO.2001.91.2.120

[12] Mayek-Pérez N, López-Castañeda C, López-Salinas E, Cumpián-Gutiérrez J, Acosta-Gallegos JA (2001) *Macrophomina phaseolina* resistance in common bean under field conditions in Mexico. Agrociencia 46: 649–661.

[13] Raguchander T, Samiyappan R, Arjunan G (1993) Biocontrol of *Macrophomina* root rot of mungbean. Indian Phytopathology 46: 379–382.

[14] De BK, Chattopadhya SB, Arjunan G (1992) Effect of potash on stem rot diseases of jute caused by *Macrophomina phaseolina* . Journal of Mycopathological Research 30: 51–55.

[15] Aly AA, Abdel-Sattar MA, Omar MR, Abd-Elsalam KA (2007) Differential antagonism of *Trichoderma sp*. against *Macrophomina phaseolina* . Journal of Plant Protection Research 47: 91–102.

[16] Crous PW, Slippers B, Wingfield MJ, Rheeder J, Marasas WFO, et al (2006) Phylogenetic lineages in the Botryosphaeriaceae. Studies in Mycology 55: 235–253. 18490983 10.3114/sim.55.1.235 PMC2104729

[17] Wrather JA, Koenning SR, Anderson TR (2003) Effect of diseases on soybean yields in the United States and Ontario (1999–2002). Plant Health Progress doi:10.1094/PHP-2003-0325-01-RV.

[18] Gupta GK, Chauhan GS (2005) Symptoms, Identification and Management of Soybean Diseases. Technical Bulletin 10 Indore, India, National Research Centre for Soybean.

[19] Mandal R, Sarkar S, Saha MN (2000) Field Evaluation of White jute (*Corchorus capsularis L*) Germplasm Against *Macrophomina phaseolina* (Tassi) Goid under Sorbhog Condition. Environment & Ecology 18: 814–818.

[20] Lanteri ML, Lamattina L, Laxalt AM (2011) Mechanisms of xylanase-induced nitric oxide and phosphatidic acid production in tomato cells. Planta 234: 845–855. 21643989 10.1007/s00425-011-1446-4

[21] Laxalt AM, Raho N, Have AT, Lamattina L (2007) Nitric oxide is critical for inducing phosphatidic acid accumulation in xylanase-elicited tomato cells. J Biol Chem 282: 21160–21168. 17491015 10.1074/jbc.M701212200

[22] Raho N, Ramirez L, Lanteri ML, Gonorazky G, Lamattina L, et al (2011) Phosphatidic acid production in chitosan-elicited tomato cells, via both phospholipase D and phospholipase C/diacylglycerol kinase, requires nitric oxide. J Plant Physiol 168: 534–539. 20951469 10.1016/j.jplph.2010.09.004

[23] Turrion-Gomez JL, Benito EP (2011) Flux of nitric oxide between the necrotrophic pathogen *Botrytis cinerea* and the host plant. Mol Plant Pathol 12: 606–616. 21722298 10.1111/j.1364-3703.2010.00695.x PMC6640425

[24] Azevedo H, Lino-Neto T, Tavares RM (2008) The Necrotroph *Botrytis cinerea* Induces a Non-Host Type II Resistance Mechanism in Pinus pinaster Suspension-Cultured Cells. Plant and Cell Physiology 49: 386–395. 18252735 10.1093/pcp/pcn015

[25] Mengiste T (2012) Plant Immunity to Necrotrophs. Annual Review of Phytopathology 50: 267–294. 10.1146/annurev-phyto-081211-172955 22726121

[26] Sahoo R, Dutta T, Das A, Sinha Ray S, Sengupta R, et al (2006) Effect of nitrosative stress on *Schizosaccharomyces pombe*: inactivation of glutathione reductase by peroxynitrite. Free Radic Biol Med 40: 625–631. 16458193 10.1016/j.freeradbiomed.2005.09.029

[27] Adak S, Bilwes AM, Panda K, Hosfield D, Aulak KS, et al (2002) Cloning, expression, and characterization of a nitric oxide synthase protein from *Deinococcus radiodurans* . Proc Natl Acad Sci U S A 99: 107–112. 11756668 10.1073/pnas.012470099 PMC117522

[28] Pant K, Bilwes AM, Adak S, Stuehr DJ, Crane BR (2002) Structure of a nitric oxide synthase heme protein from *Bacillus subtilis* . Biochemistry 41: 11071–11079. 12220171 10.1021/bi0263715

[29] Hong IS, Kim YK, Choi WS, Seo DW, Yoon JW, et al (2003) Purification and characterization of nitric oxide synthase from *Staphylococcus aureus* . FEMS Microbiol Lett 222: 177–182. 12770704 10.1016/S0378-1097(03)00254-4

[30] Golderer G, Werner ER, Leitner S, Grobner P, Werner-Felmayer G (2001) Nitric oxide synthase is induced in sporulation of *Physarum polycephalum* . Genes Dev 15: 1299–1309. 11358872 10.1101/gad.890501 PMC313797

[31] Messner S, Leitner S, Bommassar C, Golderer G, Grobner P, et al (2009) *Physarum* nitric oxide synthases: genomic structures and enzymology of recombinant proteins. Biochem J 418: 691–700. 19046139 10.1042/BJ20080192 PMC2677215

[32] Corpas FJ, Barroso JB, Carreras A, Quiros M, Leon AM, et al (2004) Cellular and subcellular localization of endogenous nitric oxide in young and senescent pea plants. Plant Physiol 136: 2722–2733. 15347796 10.1104/pp.104.042812 PMC523336

[33] Valderrama R, Corpas FJ, Carreras A, Fernandez-Ocana A, Chaki M, et al (2007) Nitrosative stress in plants. FEBS Lett 581: 453–461. 17240373 10.1016/j.febslet.2007.01.006

[34] Rodriguez-Serrano M, Romero-Puertas MC, Zabalza A, Corpas FJ, Gomez M, et al (2006) Cadmium effect on oxidative metabolism of pea (*Pisum sativum L*.) roots. Imaging of reactive oxygen species and nitric oxide accumulation in vivo. Plant Cell Environ 29: 1532–1544. 16898016 10.1111/j.1365-3040.2006.01531.x

[35] Madeo F, Frohlich E, Ligr M, Grey M, Sigrist SJ, et al (1999) Oxygen stress: a regulator of apoptosis in yeast. J Cell Biol 145: 757–767. 10330404 10.1083/jcb.145.4.757 PMC2133192

[36] Teather RM, Wood PJ (1982) Use of Congo red-polysaccharide interactions in enumeration and characterization of cellulolytic bacteria from the bovine rumen. Appl Environ Microbiol 43: 777–780. 7081984 10.1128/aem.43.4.777-780.1982 PMC241917

[37] Mandels M, Hontz L, Nystrom J, Lee RLIB (2010) Enzymatic hydrolysis of waste cellulose. Biotechnol Bioeng. Vol. XVI, pages 1471–93 (1974). Biotechnol Bioeng 105: 3–25 discussion 21–22. 19937801 10.1002/bit.22603

[38] Tseng M-J, Yap M-N, Ratanakhanokchai K, Kyu KL, Chen S-T (2002) Purification and characterization of two cellulase free xylanases from an alkaliphilic *Bacillus firmus* . Enzyme and Microbial Technology 30: 590–595.

[39] Griess P (1879) Bemerkungen zu der abhandlung der H.H. Weselsky und Benedikt “Ueber einige azoverbindungen”. Chemische Berichte 12: 426–428.

[40] Tamura K, Peterson D, Peterson N, Stecher G, Nei M, et al (2011) MEGA5: molecular evolutionary genetics analysis using maximum likelihood, evolutionary distance, and maximum parsimony methods. Mol Biol Evol 28: 2731–2739. 21546353 10.1093/molbev/msr121 PMC3203626

[41] Edgar RC (2004) MUSCLE: a multiple sequence alignment method with reduced time and space complexity. BMC Bioinformatics 5: 113. 15318951 10.1186/1471-2105-5-113 PMC517706

[42] Bailey LT, Elkan C (1994) Fitting a mixture model by expectation maximization to discover motifs in biopolymers. In: Altman R, Brutlag D, Karp P, Lathrop R, Searls D, editors. Proceedings of the Second International Conference on Intelligent Systems for Molecular Biology. Menlo Park, California: AAAI Press. 28–36. 7584402

[43] Hunter S, Jones P, Mitchell A, Apweiler R, Attwood TK, et al (2012) InterPro in 2011: new developments in the family and domain prediction database. Nucleic Acids Res 40: D306–312. 22096229 10.1093/nar/gkr948 PMC3245097

[44] Carlberg I, Mannervik B (1975) Purification and characterization of the flavoenzyme glutathione reductase from rat liver. J Biol Chem 250: 5475–5480. 237922

[45] Aebi H (1984) Catalase in vitro. Methods Enzymol 105: 121–126. 6727660 10.1016/s0076-6879(84)05016-3

[46] Miyake C, Asada K (1992) Thylakoid-Bound Ascorbate Peroxidase in Spinach Chloroplasts and Photoreduction of Its Primary Oxidation Product Monodehydroascorbate Radicals in Thylakoids. Plant and Cell Physiology 33: 541–553.

[47] Saville B (1958) A scheme for the colorimetric determination of microgram amounts of thiols. Analyst 83: 670–672.

[48] Crane BR, Sudhamsu J, Patel BA (2010) Bacterial nitric oxide synthases. Annu Rev Biochem 79: 445–470. 20370423 10.1146/annurev-biochem-062608-103436

