# Supplementary figures and images for: Nitric Oxide Production by Necrotrophic Pathogen Macrophomina phaseolina and the Host Plant in Charcoal Rot Disease of Jute: Complexity of the Interplay between Necrotroph–Host Plant Interactions
Source: PLoS One. 2014 Sep 10;9(9):e107348. doi: 10.1371/journal.pone.0107348 (PMC4160249; doi:10.1371/journal.pone.0107348)

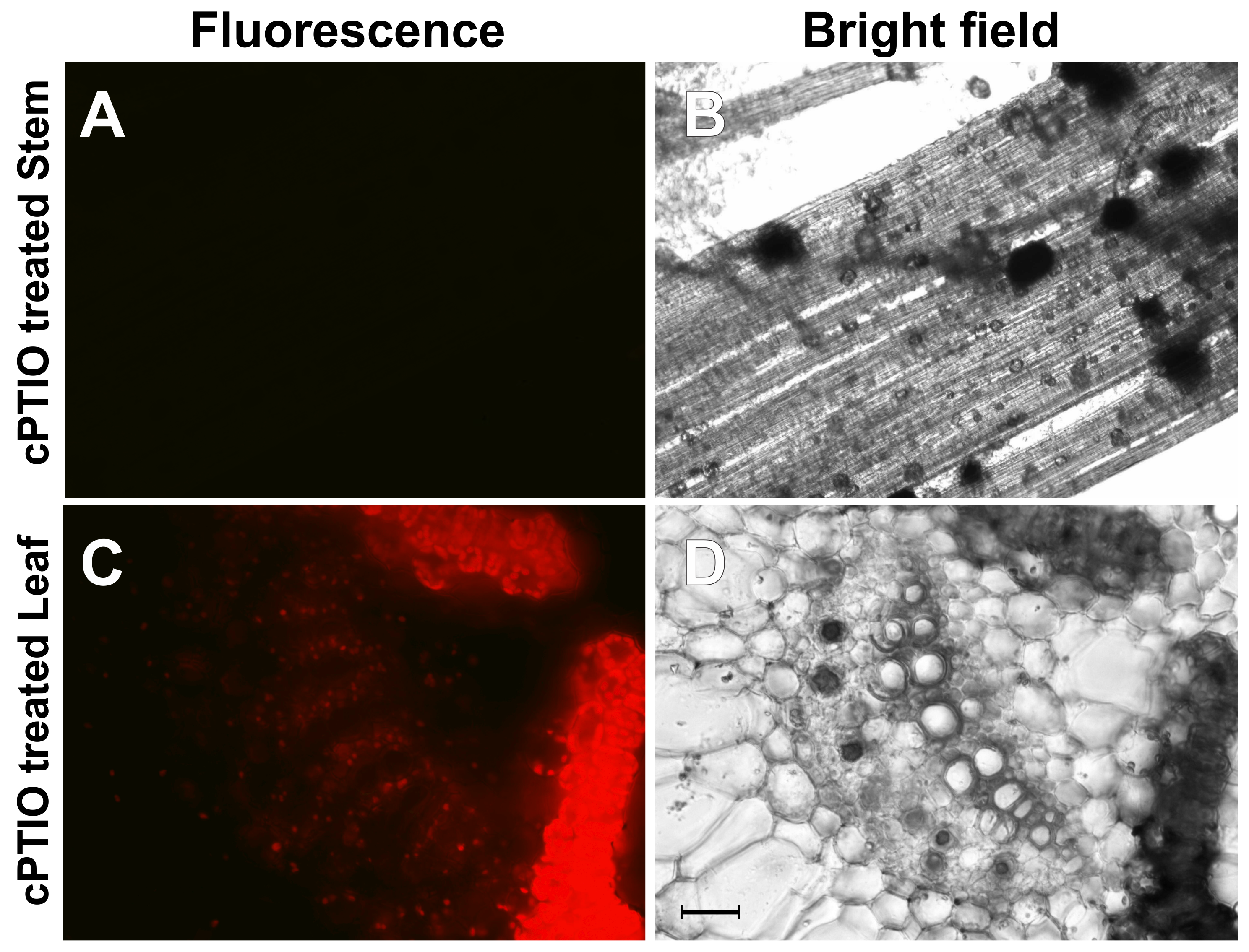

Supplement: Figure S1 — Control experiment with NO scavenger cPTIO. Representative images illustrate the scavenging of NO by cPTIO in C. capsularis stem and leaf. Stem section (A), leaf section (C) pre-incubated with the NO scavenger cPTIO (200 µM) (negative control). Corresponding bright fields are (B) and (D). The orange yellow color corresponds to the chlorophyll autofluorescence. Figures are representative of at least six independent experiments. Bar = 250 µm. (TIF) [file pone.0107348.s001.tif]

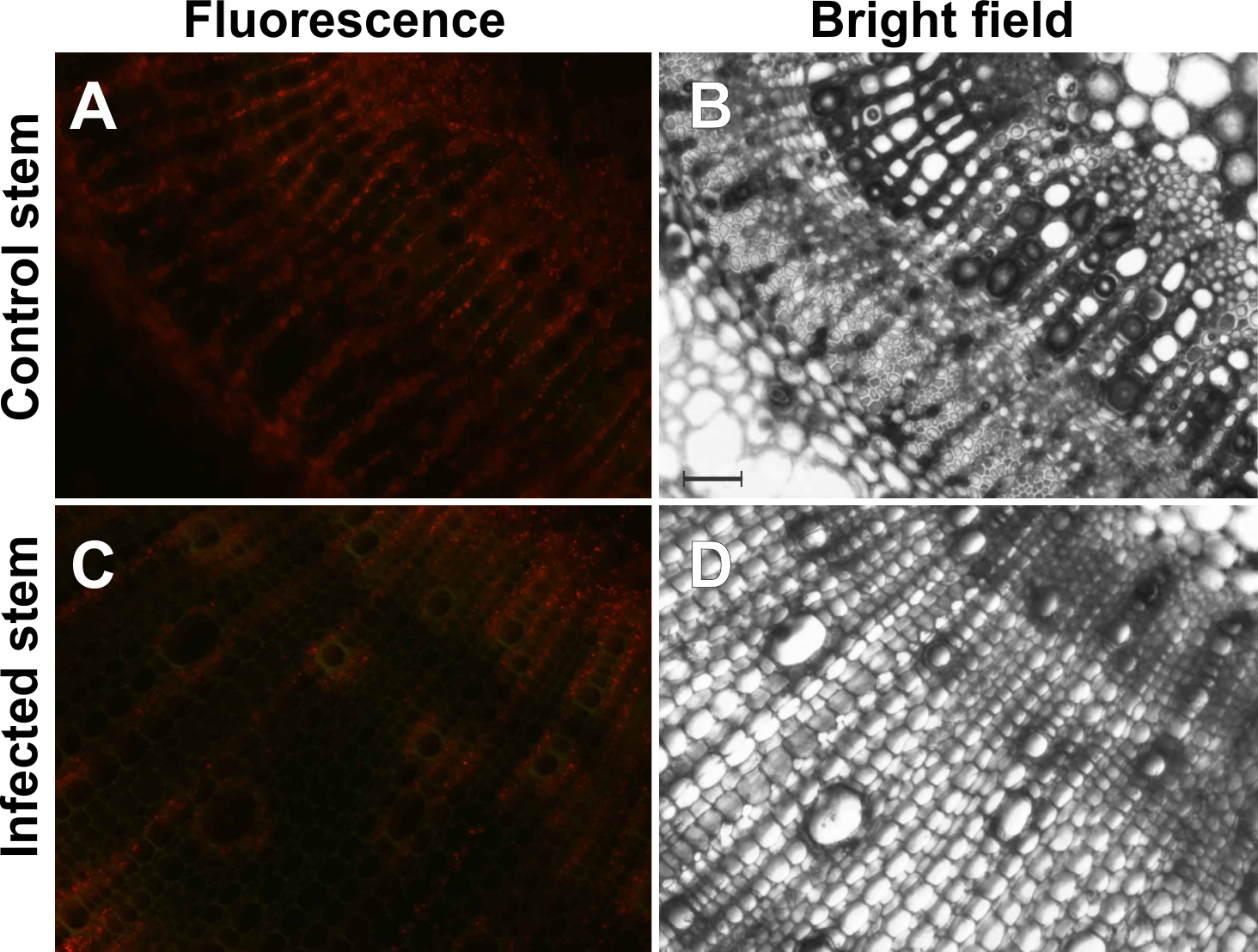

Supplement: Figure S2 — Fluorescence microscopic images of control and infected jute stem sections for ROS detection. (A) and (C) represent control and infected stem sections respectively. (B) and (D) are the corresponding bright field Images of (A) and (C) respectively. No ROS specific fluorescence was observed in all the sections. The red colour corresponds to the autofluorescence. Figures are representative of at least six independent experiments. Bar = 250 µm. (TIF) [file pone.0107348.s002.tif]

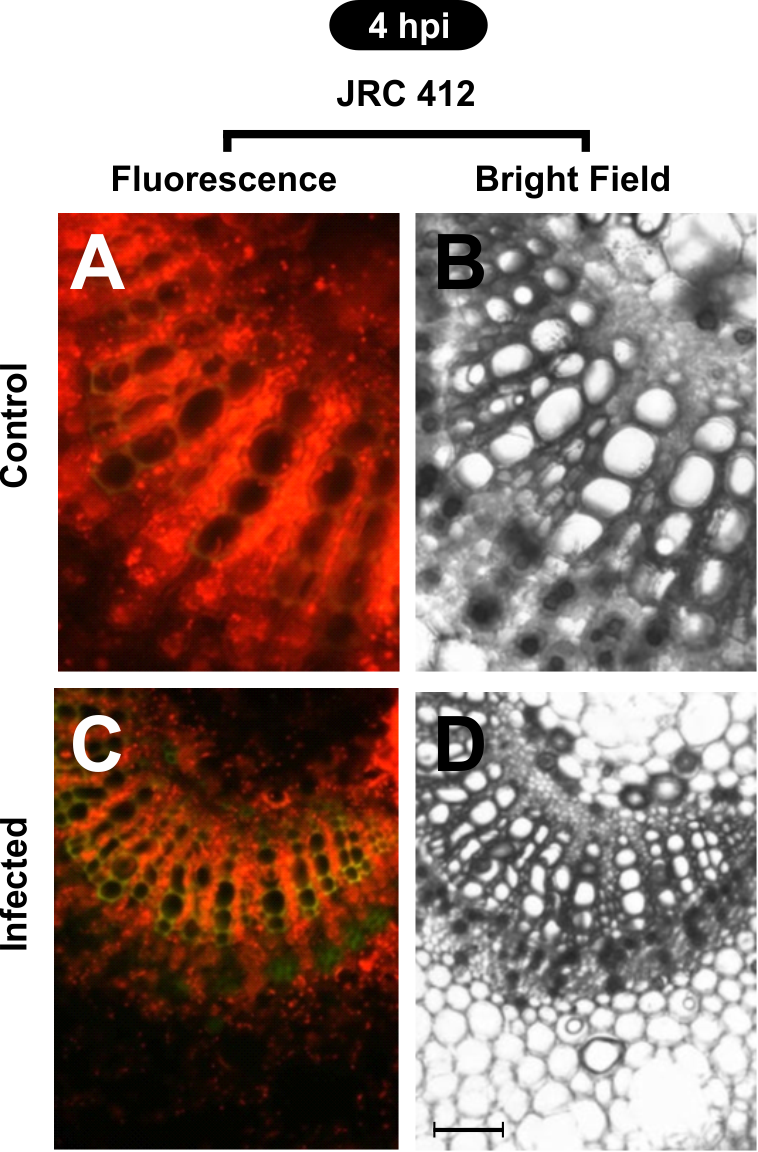

Supplement: Figure S3 — Time dependent NO detection in control and infected jute leaf by fluorescence microscopy. The cross sections of control jute stem showed no NO specific fluorescence (A). NO specific green fluorescence was detected in the cross sections of infected jute stem after 4 hour post inoculation (4 hpi)(C). (B) and (D) are the corresponding bright field images of (A) and (C) respectively. The red colour corresponds to the autofluorescence. Bar = 250 µm. (TIF) [file pone.0107348.s003.tif]

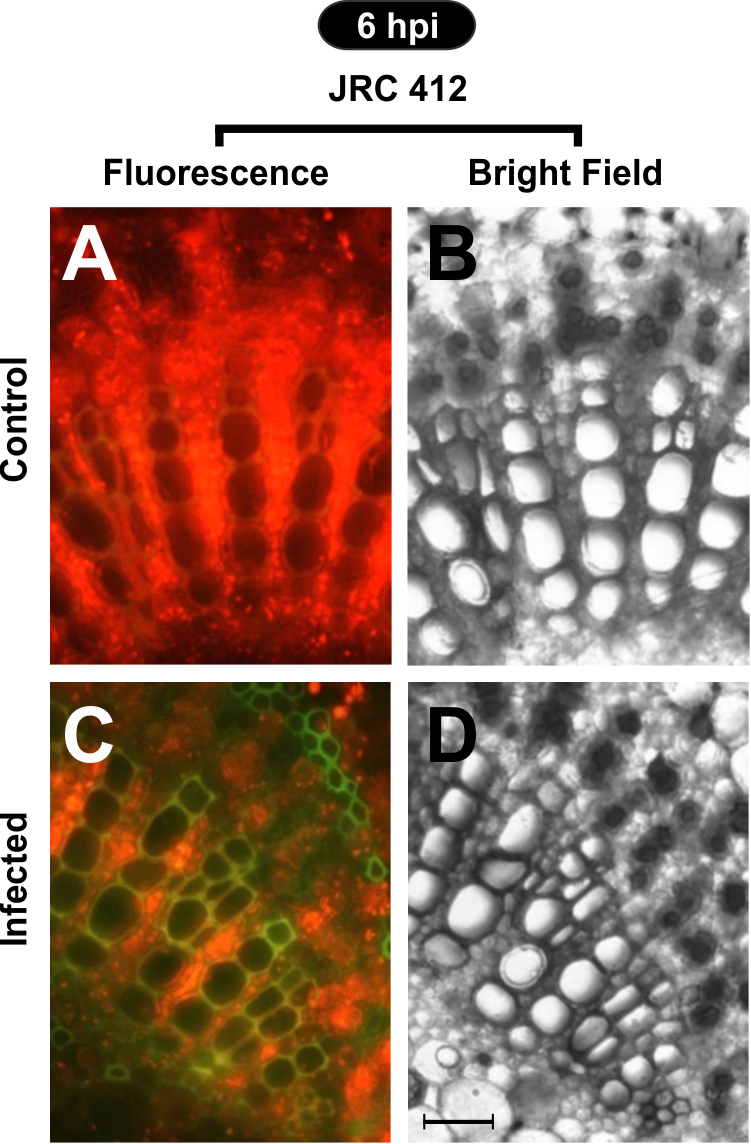

Supplement: Figure S4 — Time dependent NO detection in control and infected jute leaf by fluorescence microscopy. The cross sections of control jute stem showed no NO specific fluorescence (A). NO specific green fluorescence was detected in the cross sections of infected jute stem after 6 hour post inoculation (6 hpi)(C). (B) and (D) are the corresponding bright field images of (A) and (C) respectively. The red colour corresponds to the autofluorescence. Bar = 400 µm. (TIF) [file pone.0107348.s004.tif]

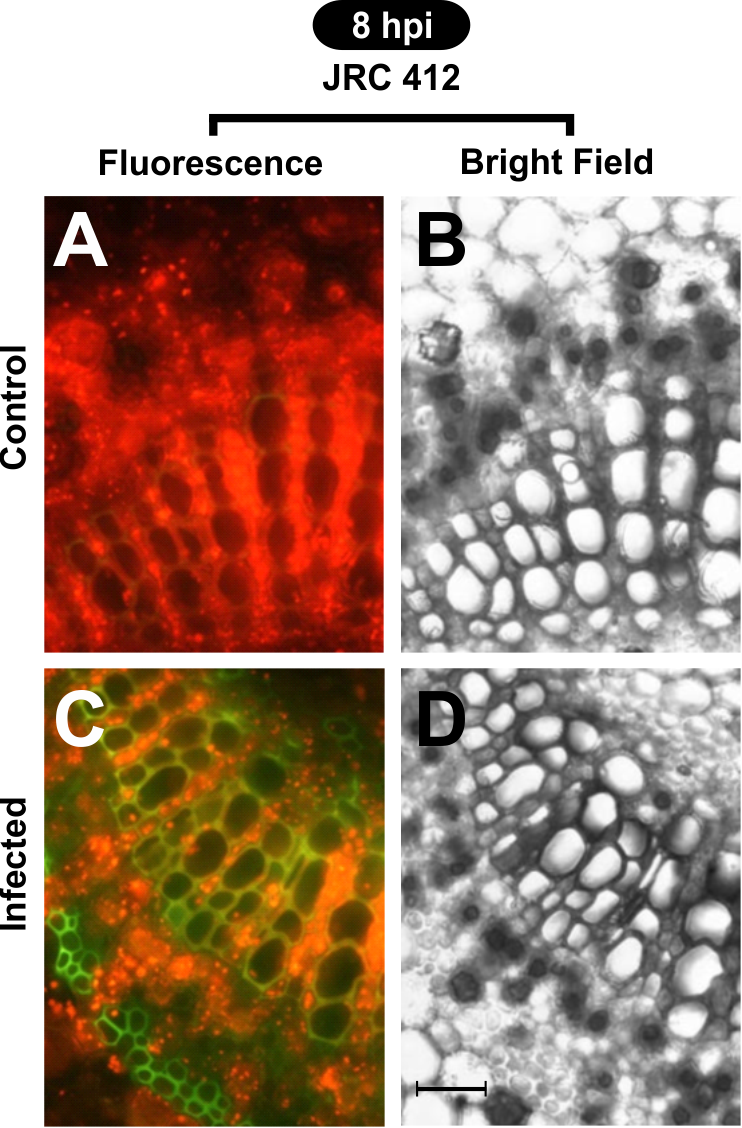

Supplement: Figure S5 — Time dependent NO detection in control and infected jute leaf by fluorescence microscopy. The cross sections of control jute stem showed no NO specific fluorescence (A). NO specific green fluorescence was detected in the cross sections of infected jute stem after 8 hour post inoculation (8 hpi) (C). (B) and (D) are the corresponding bright field images of (A) and (C) respectively. The red colour corresponds to the autofluorescence. Bar = 400 µm. (TIF) [file pone.0107348.s005.tif]

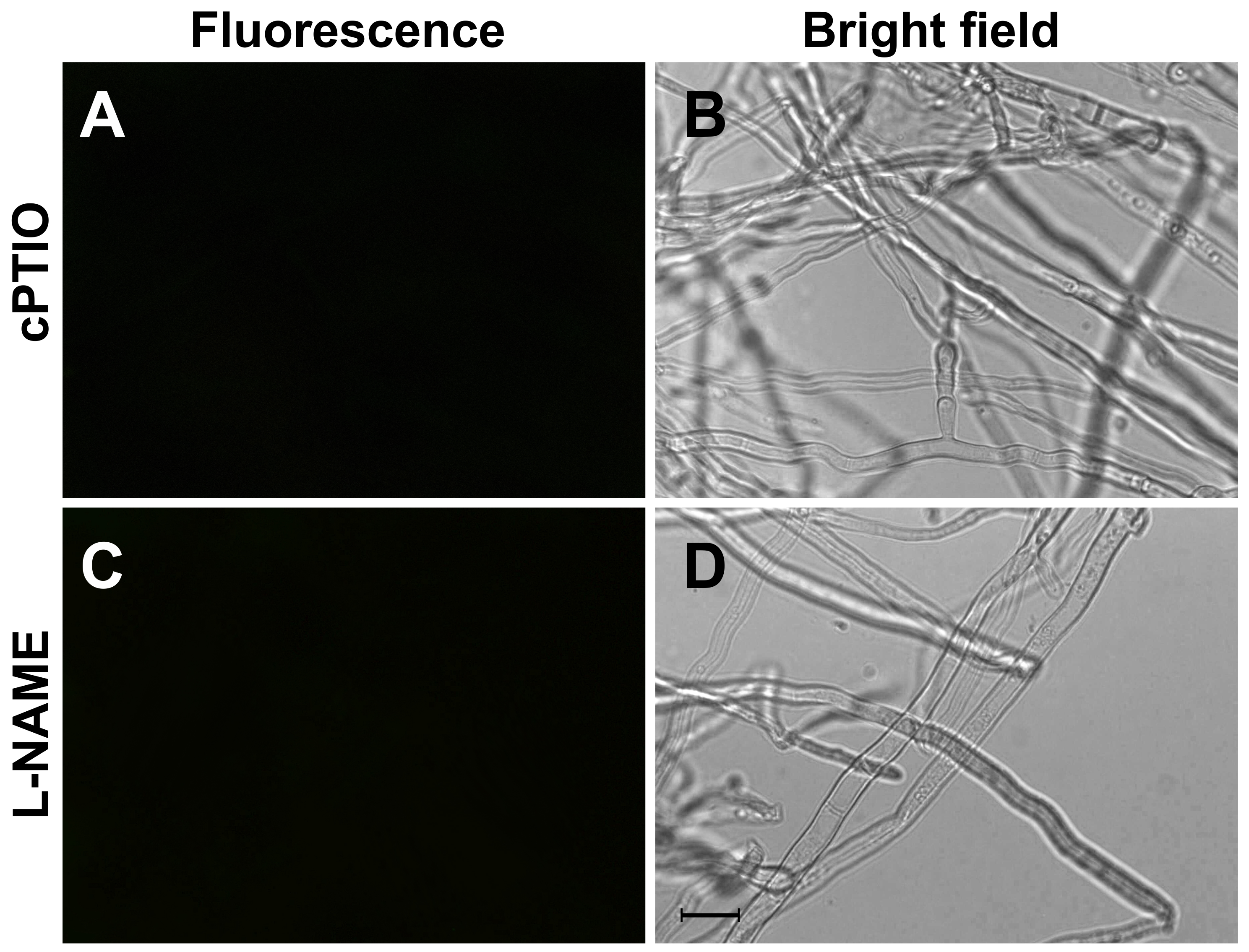

Supplement: Figure S6 — Control experiment with NO scavenger cPTIO and L-NAME. Representative images illustrate the effect of cPTIO and L-NAME in M. phaseolina. Fungal mycelia were pre incubated with the NO scavenger cPTIO (200 µM) Panel A and with L-NAME (Panel C). Absence of bright green fluorescence indicated the specificity of NO production. Corresponding bright fields are (B) and (D). Figures are representative of at least six independent experiments. Bar = 30 µm. (TIF) [file pone.0107348.s006.tif]

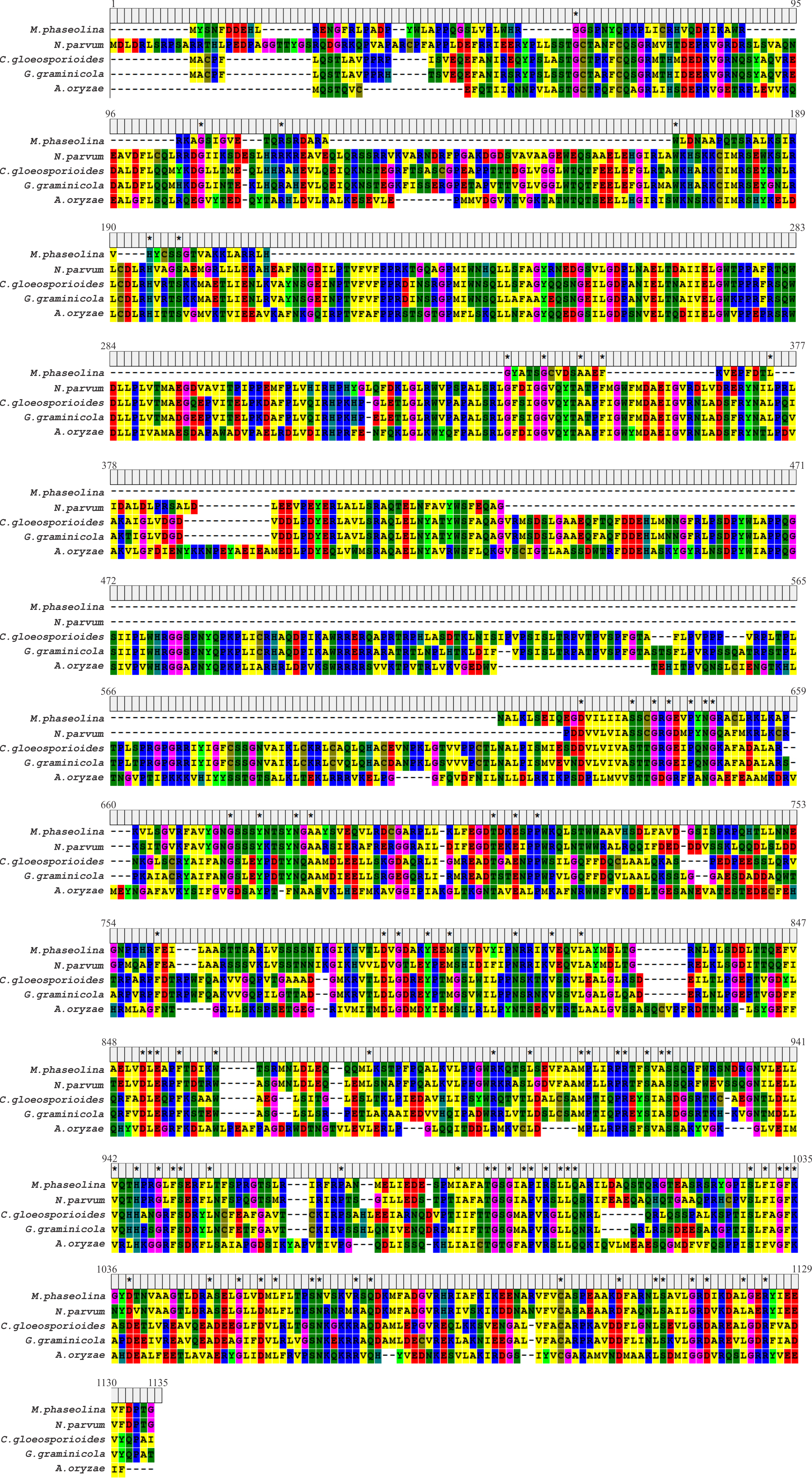

Supplement: Figure S7 — Multiple sequence alignment of the reductase domain of M. phaseolina MS6 with the four fungal NOS sequences. Five fungal NOS sequences of the following species Macrophomina phaseolina, Neofusicoccum parvum, Colletotrichum gloeosporioides, Glomerella graminicola, Aspergillus oryzae were used using MUSCLE algorithm in MEGA 5. The asterisks in the sequence positions denote the exact match of amino acids in all 5 sequences. (TIF) [file pone.0107348.s007.tif]
